# Is publishing gold open-access worth it? An assessment of hybrid and gold open-access publishing models of medical journals on their impact

**DOI:** 10.12688/f1000research.159550.1

**Published:** 2024-12-09

**Authors:** Abrar K. Thabit, Noha I. Ashy, Abdulrahman O. Fallatah, Ali S. Alquzi

**Affiliations:** 1 Pharmacy Practice Department, Faculty of Pharmacy, King Abdulaziz University, Jeddah, Saudi Arabia; 2 Faculty of Pharmacy, King Abdulaziz University, Jeddah, Saudi Arabia

**Keywords:** publishing, hybrid, open-access, publisher, journal, journal impact factor

## Abstract

**Background:**

Hybrid and gold open-access (OA) are the most common publishing models. The latter requires fees to allow full-text visibility upon publishing, whereas hybrid journals offer the option to publish gold OA or for free (subscription-based) where only users with access can get the full-text. We aimed to evaluate the impact of the publishing model and other factors on medical journals’ impact.

**Methods:**

A sample of hybrid and gold OA medical journals indexed in Web of Science (WOS) and Scopus were included. The effect of the publishing model and other factors on journals’ impact factor (IF), CiteScore, quartile, and number of citations was assessed.

**Results:**

402 journals were included, 201 in each group. Hybrid and gold OA journals had a median age of 32 and 21 years, respectively ( *P*<0.001). The median publishing cost in gold OA journals was $2,690, and 46.3% of them publish continuously. Publishing model, journal’s age, being of an organization/society, and EMBASE indexation didn’t affect IF, CiteScore, number of citations, and WOS quartile ( *P*>0.05). However, gold OA model wasn’t significantly associated with Q1 ranking in Scopus (OR, 0.49; 95% CI, 0.25-0.94; *P*=0.032), which indicates that hybrid journals were more likely to have such ranking.

**Conclusion:**

These findings indicate that gold OA publishing doesn’t necessarily result in higher impact, which contradicts the claim that such model enhances citations. Therefore, authors can continue to publish in hybrid journals without being concerned about getting cited. Gold OA journals are encouraged to reduce their fees to facilitate global research access.

## Introduction

Open access (OA) publications allow individuals to read published scientific articles for free, regardless of location, institutional affiliation, or subscription to the journal (or its publisher). ^1^ Currently, OA publishing model includes several subtypes, the most common type is gold OA, where authors retain the copyright to their published article that is made freely available to any user in the online journal. ^2^ Gold OA publishing model requires the payment of a certain fee by the authors. The other type of OA publishing model is green open access, which permits the placement of the authors’ manuscript in an institutional repository and then the article becomes freely available after an embargo period set by the journal. Green OA journals usually ask for page charges, in which the total is usually lower than gold OA charges. The concept of gold OA publishing was recently adapted within the last 20 years as journals used to be subscription-based only (i.e., access to articles are only allowed to users subscribed to the journal via their institution or a personal subscription while authors publish their work without a fee). ^3^ However, upon the introduction of gold OA concept, it became widespread among publishers as numerus journals became hybrid, meaning that they allow both subscription-based publishing for authors opting to publish for free and gold OA publishing for authors willing to pay the publication fees. OA publishing is becoming more popular as large research funding agencies, such as the National Institutes of Health in the United States and the Bill and Melinda Gates Foundation, require researchers to make their work freely available. ^2, 4^

Access to medical literature is an essential part of healthcare professionals’ job to make decisions regarding disease management and implementation of certain interventions. Instead of attempting to base their decisions simply on the subset of research to which they have access, clinicians can make more informed health decisions when they have entire access to studies relevant to the cases they are treating. ^5^ OA articles are more frequently read, and hence promote access to knowledge and new advances in different disciplines. Since the early 1990s, there has been a notable surge in OA publication, with an estimated annual growth in published articles of 30%. ^6^ Furthermore, a number of universities and journal publishers have recently signed Open Access Transformative Agreements (OATAs), which require a greater transition to OA article publication over a predetermined time period, in response to recommendations from the research communities, such as the Efficiency and Standards for Article Charges Initiative. ^7^

Despite the advantages of OA publishing, it has a major disadvantage that should be acknowledged. To publish an OA article, journals ask the publishing fees, which are commonly known as article processing charges (APCs). These charges, which sometimes reach thousands of US dollars, may prevent authors with limited institutional or personal funds from publishing openly. ^8, 9^ The unaffordability of APCs may create a barrier to sharing research. ^10^ Such major issue influenced many researchers to call for free access to research without barriers (i.e., the need for subscription or forcing authors to pay APCs to make their research accessible to all. ^11^

The prevailing view suggests that the advent of OA publishing model enhances the spread of knowledge and potentially increases citation counts for authors; though, this continues to be contested. Therefore, the objective of this study is to evaluate the effect of the gold OA publishing model on several journals’ metrics on Web of Science (WOS) and Scopus by comparing it to hybrid publishing model.

## Methods

### Journals selection

Medical journals indexed in Science Citation Index Expanded (SCIE) of WOS and Scopus that are fully published in English were eligible for inclusion in the study. While journals that publish both clinical and basic sciences studies were included, journals that only publish articles in basic sciences, ethics, healthcare systems management, administration, and policy were excluded. Journals were divided into two groups based on their publishing model as either hybrid or gold OA.

### Definitions

• Hybrid journals: Journals that offer subscription-based and gold OA publishing options. Subscription-based publishing means that only users with access to the journal through an institutional or a personal subscription to the journal can get the full-text of the published articles.
• Gold OA journals: Journals that require authors to pay publishing fees that’s commonly known as APC to have their articles fully visible and available for download to anyone immediately upon publishing.
• Impact factor (IF): 2-year IF of 2021-2022. It’s calculated based on the number of citations of all the articles published in the journal within the last two years.
• CiteScore: 3-year CiteScore of 2019-2022. It’s calculated based on the number of citations of all the articles published in the journal within the last three years.
• Number of citations in WOS: The total number of times articles published in the journal were cited since journal’s inception.
• Number of citations in Scopus: The total number of times articles published in the journal were cited between 2019-2022.
• Quartile: The quartile in which the journal is ranked, where Q1 involves the journals that are ranked the top 25% within a certain specialty, Q2 involves the journals that are ranked between 26-50%, Q3 involves the journals that are ranked between 51-75%, and Q4 involves the journals that are ranked between 76-100%. If a journal is ranked in more than one specialty, the average ranking was calculated.

The IF, CiteScore, number of citations, and quartile of each journal were collected from the journals’ records on Journal Citation Reports and Scopus.

### Statistical analysis

Categorical data were compared using Chi-square test and were presented as numbers and percentages, whereas continuous data were compared using Mann-Whitney test and presented using median [interquartile range (IQR)] as they were deemed skewed based on results of Shapiro-Wilk test of normality. Linear regression was conducted to assess the effect of different factors on IF and CiteScore. Multinomial regression was used to evaluate the association of different factors with Q1 ranking in WOS and Scopus. Odds ratios (OR) and 95% confidence intervals (CI) were reported. Fitting of the regression models was assessed using goodness-of-fit test. Omnibus test of significance was used to assess the significance of the regression models. A *P* value < 0.05 was deemed statistically significant. Analyses were performed on SPSS version 24.0 (IBM Corp., Armonk, NY, USA).

## Results

A total of 402 journals included, 201 in each group. Table 1 shows the characteristics of the included journals. Hybrid and gold OA journals had a median [IQR] age of 32 [23–41.5] and 21 [13–28] years, respectively ( *P* < 0.001). Most of the journals were international (39.3% vs. 53.7%; *P* < 0.001). In terms of volumes and issues, hybrid journals publish more frequently per year than gold OA journals (8 vs. 6; *P* = 0.041). However, 93 (46.3%) of the gold OA publish continuously; thus, were not included in the calculation of the median. More than half of the gold OA journals publish both clinical and basic sciences studies (35.3% vs. 53.2%; *P* < 0.001). The median [IQR] cost of publication in gold OA journals was 2,690 [2,000–2,990]. Although a significant difference was observed in terms of IF between the hybrid and the gold OA publishing models (2.90 [2.20–4.15] vs. 3.30 [2.40–4.45]; *P* = 0.021), no statistically significant difference was observed in terms of CiteScore, number of citations, and quartiles in both WOS and Scopus ( *P* > 0.05 for all comparisons).

**Table 1.** Characteristics of the journals based on their publishing model.

Characteristic	Hybrid (n=201)	Gold Open-access (n=201)	*P* value
Journal age (years)	32 [23–41.5]	21 [13–28]	< 0.001
Publisher ^*^			< 0.001
Springer	29 (14.4)	48 (23.9)	
Elsevier	38 (18.9)	14 (7)	
Wiley	25 (12.4)	19 (9.4)	
Taylor & Francis	23 (11.4)	13 (6.5)	
Sage	11 (5.5)	18 (9)	
Lippincott Williams & Wilkins	17 (8.5)	1 (0.5)	
Cambridge	7 (3.5)	2 (1)	
Other	51 (25.4)	86 (42.7)	
Journal’s country ^*^			< 0.001
International	79 (39.3)	108 (53.7)	
USA	65 (32.3)	31 (15.4)	
UK	16 (8)	12 (6)	
Canada	10 (5)	4 (2)	
Japan	5 (2.5)	5 (2.5)	
Other	26 (12.9)	41 (20.4)	
Specialty ^*^			< 0.001
General medicine	20 (10)	32 (15.9)	
Neurology/Neurosurgery	18 (9)	11 (5.5)	
Oncology	15 (7.4)	14 (7)	
Cardiology	12 (6)	17 (8.4)	
Endocrinology	13 (6.4)	14 (7)	
Other	123 (61.2)	113 (56.2)	
Publication frequency (per year) ^**^	8 [6–12]	6 [4–12]	0.041
Journal published both clinical and basic sciences	71 (35.3)	107 (53.2)	< 0.001
Publication cost ($)	-	2,690 [2,000–2,990]	-
Year journal switched to gold OA (n=124) ^***^	-	2014 [2008–2019]	-
Journal belongs to an organization	109 (54.2)	96 (47.8)	0.195
Impact factor	2.9 [2.2–4.2]	3.3 [2.4–4.5]	0.021
Quartile in WOS			0.163
Q1	59 (29.4)	52 (25.6)	
Q2	59 (29.4)	81 (40.3)	
Q3	56 (27.9)	48 (23.90	
Q4	27 (13.4)	20 (10)	
Number of citations in WOS	4,918 [2,510–8,648]	3,963 [2,201–8,915]	0.394
CiteScore	5.10 (3.60–6.85)	5 (3.55–7.10)	0.951
Quartile in Scopus			
Q1	116 (57.7)	88 (43.8)	
Q2	64 (31.8)	83 (41.3)	
Q3	21 (10.4)	30 (14.9)	
Number of citations in Scopus	2,420 [1,325–4,521]	2,972 [1,307–6,486]	0.060
Indexed in EMBASE	188 (93.5)	181 (90)	0.203

Data are presented as median [interquartile range] or n (%)

OA, open-access; WOS, Web of Science.

^*^ Only top 5 were listed.

^**^ n=93 of gold open-access journals publish continuously (i.e., without assigning articles to a volume or issue); hence, not included in this median.

^***^ The remaining 77 journals were open-access since inception.

When different factors were assessed for their effect on IF, only publishing both clinical and basic science studies was significantly associated with higher IF (OR, 2.03; 95% CI,1.19–3.45; *P* = 0.010). The same factor was associated with higher CiteScore (OR,3.63; 95% CI, 1.43–9.20; *P* = 0.007). On the other hand, the publishing model (hybrid vs. gold OA) did not influence IF nor CiteScore (OR, 1.38; 95% CI, 0.81–2.36; *P* = 0.237 and OR,0.81; 95% CI, 0.32–2.04; *P* =0.648, respectively). The effect of different factors on IF and CiteScore are shown in Tables 2 and 3, respectively.

**Table 2.** Effect of different factors on impact factor.

Factor	β Coefficient ± SE	OR (95% confidence interval)	*P* value
Publishing model			0.234
Hybrid	Ref	Ref	
Gold open-access	0.32 ± 0.27	1.38 (0.81–2.36)	
Journal’s age	-0.001 ± 0.007	1 (0.99–1.01)	0.922
Journal published both clinical and basic sciences	0.71 ± 0.27		0.010
Journal belongs to an organization	0.14 ± 0.26	1.15 (0.69–1.91)	0.597
Indexed in EMBASE	0.62 ± 0.48	1.86 (0.72–4.81)	0.200

**Table 3.** Effect of different factors on CiteScore.

Factor	β Coefficient ± SE	OR (95% confidence interval)	*P* value
Publishing model			0. 648
Hybrid	Ref	Ref	
Gold open-access	-0.22 ± 0.47	0.81 (0.32–2.04)	
Journal published both clinical and basic sciences	1.29 ± 0.47	3.63 (1.43–9.2)	0.007
Journal belongs to an organization	0.05 ± 0.46	1.05 (0.43–2.56)	0.921
Indexation in EMBASE	0.79 ± 0.84	2.21 (0.42–11.56)	0.347
Journal’s age	0 ± 0.01	1 (0.98–1.02)	0.982

The effect of different factors on the quartiles of the journals in WOS was evaluated, where none of the factors was associated with ranking in either quartile. Table 4 lists the factors and their effect on ranking in Q1 of WOS. On the other hand, hybrid journals were associated with ranking in Q1 of Scopus as the odds of gold OA journals being ranked as Q1 was less than one (OR, 0.49; 95% CI 0.25–0.94; *P* = 0.032). Table 5 lists the factors and their effect on ranking in Q1 of Scopus.

**Table 4.** Effect of different factors on Q1 ranking in Web of Science.

Factor	OR (95% confidence interval)	*P* value
Publishing model		
Hybrid	Ref	
Gold open-access	1.19 (0.58–2.43)	0.638
Journal published both clinical and basic sciences	1.06 (0.52–2.16)	0.870
Journal belongs to an organization	1.29 (0.65–2.58)	0.468
Indexation in EMBASE	0.38 (0.04–3.33)	0.384
Journal’s age	1.00 (0.99–1.02)	0.914

**Table 5.** Effect of different factors on Q1 ranking in Scopus.

Factor	OR (95% confidence interval)	*P* value
Publishing model		
Hybrid	Ref	
Gold open-access	0.49 (0.25–0.94)	0.032
Journal published both clinical and basic sciences	0.69 (0.36–1.31)	0.257
Journal belongs to an organization	1.50 (0.79–2.83)	0.214
Indexation in EMBASE	< 0.0001 (< 0.0001–< 0.0001)	< 0.0001
Journal’s age	0.99 (0.97–1.00)	0.079

A subgroup analysis in the gold OA group to assess the effect of publishing cost on ranking in WOS showed that higher APCs were associated with Q3 ranking (OR, 2.76; 95% CI, 1.22–6.23; *P* = 0.015) and negatively associated with Q1 ranking (OR, 0.57; 95% CI, 0.26–1.23; *P* < 0.001), but no effect was observed with Q2 ranking. The same observation was found with Q1 ranking in Scopus, where journals with high APCs were less likely to be ranked in Q1 (OR, 0.25; 95% CI, 0.11–0.54; *P* < 0.001).

## Discussion

This is the first study to evaluate the impact of gold OA publishing model on journals’ metrics. Findings from this study suggest that gold OA publishing model does not have an impact on IF or CiteScore despite the significant difference in IF, but not CiteScore, between hybrid and gold OA journals. These results align with findings from previous studies that explored similar themes, indicating that while OA models aim to increase accessibility, they do not necessarily confer a citation advantage over hybrid models. ^12, 13^ For instance, some studies also found minimal differences in citation metrics between different publishing models, suggesting that other factors, such as journal reputation and article quality, might play more pivotal roles in influencing these metrics. ^14– 16^ A study by Chua et al found a higher number of citations of OA journals that did not correlate with IF (Spearman’s rho = 0.187; *P* = 0.60). ^16^ Similarly, the increment increase in IF with increased citations was minimal and not statistically significant (β coefficient = 3.35; 95% CI -0.464–7.156; *P* = 0.084). ^16^ As such, it is argued that the very high fees required by gold OA journals (reaching a median [IQR] of $2,690 [2,000–2,990] and a range of $75–5,460) are not justified when considering that many researchers call that science should be free to access by everyone. Therefore, these journals should consider lowering fees to cover necessary editorial processing and offer waivers for unfunded authors provided they provide a proof of lack of funds regardless of their country of origin (as many gold OA journals already offer waivers or discounts for authors from low-income countries). This approach aligns with the ethical imperative to democratize access to scientific knowledge, a sentiment echoed in the literature. ^14– 16^

Gold OA publishing model is a relatively new concept in research publishing as seen with the significant difference in the median [IQR] ages of hybrid and gold OA journals (32 [23–41.5] vs. 21 [13–28]; *P* < 0.001). Nonetheless, it become widespread, especially as many journals that were formerly hybrid have transitioned to gold OA, where the median [IQR] year of switching was 2014 [2008–2019]. This transition is indicative of the broader shift in the publishing landscape towards more open access models, a trend noted in earlier studies. ^14, 15^ Despite the significant difference in age, it is noteworthy that gold OA journals reached a median total number of citations in WOS since journals’ inception that was not statistically significant from that of hybrid journals, though it was numerically lower (3,963 vs. 4,918; *P* = 0.351). This could be possibly attributed to the large number of gold OA journals (n=93 of 201; 46.3%) that publish articles continuously without assigning them to a certain volume or issue, unlike hybrid journals that have limited quotas. This observation resonates with previous reports that showed that continuous publication models might influence citation patterns differently compared to traditional issue-based models. ^14, 15^

The lack of difference on the median total number of citations on Scopus reported for the years 2019–2022 contradicts the hypothesis that gold OA could achieve a high impact within a short period, given that Scopus reports citations within a specific time frame rather than a lifetime total, as is the case with WOS. This discrepancy highlights the complexity of evaluating impact across different databases per previous reports. ^14, 15^

Journals that publish using only gold OA model market themselves to researchers looking to gain more citations and broader readership. ^17^ Nevertheless, and contrary to that claim, the current study found no significant difference in the number of citations between gold OA and hybrid publishing models. Additionally, were observed more frequently with hybrid than with gold OA journals, while no difference was noted in the distribution of hybrid and gold OA journals among the different WOS quartiles. This outcome suggests that the prestige and perceived impact of hybrid journals may still hold sway in the academic community. ^14, 15^ Moreover, in their studies, Gargouri, et al and McCabe, et al have emphasized the importance of publishing both basic science and clinical research to reach a broader audience. The ability of journals that encompass a wide range of disciplines to appeal to a diverse readership has been highlighted as a significant factor in increasing a journal’s impact. ^14, 15^ In the current study, this notion was supported by the observation that hybrid journals, which often publish a mix of basic and clinical research, were more likely to achieve higher quartile rankings in Scopus.

These results of the current study suggest that unfunded researchers who cannot afford gold OA publishing should be less concerned about the citation impact of their work. It is worth noting that while articles published in subscription-based hybrid journals may not be freely accessible, readers can still obtain the full text by contacting the corresponding author via email or through researchers platforms like ResearchGate and Academia.edu. ^18^ This highlights the role of author accessibility in ensuring the wider dissemination of research findings, regardless of the publishing model. Nevertheless, the current study highlights the importance of considering the call for free access to scientific information. To promote equitable access, gold OA journals should offer lower fees and waivers for unfunded authors, regardless of their country of origin. By reducing financial barriers, these journals can facilitate the dissemination of research and contribute to the broader scientific community.

It is important to acknowledge the strengths and limitations of this study. One of the strengths is that it is the first to directly compare the impact of hybrid and gold OA journals. By including a large random sample of journals and meeting the target sample size, the study provides a comprehensive analysis of the two models. It also evaluated several factors for their potential association with IF of WOS, CiteScore of Scopus, and quartiles of both databases. However, certain limitations should be considered. The study did not evaluate the impact of gold OA articles published in hybrid journals, which could potentially influence the overall impact comparison. Unfortunately, such an assessment was not feasible due to the longstanding existence of many hybrid journals. Additionally, journals that offer green OA, where articles become fully open access after an embargo period by paying page charges, were not included in the current study given their limited number, which could have made the comparison with the abundant hybrid and gold OA unjustified. Future research could explore the impact of these factors on citation metrics and readership. Further research is also warranted to investigate other aspects of OA publishing. While this study focused on IF and CiteScore, future studies could explore additional metrics such as altmetrics, which capture online attention and engagement with scholarly articles. Moreover, qualitative research methods could be employed to understand the perceptions and experiences of researchers regarding hybrid and gold OA publishing models. This would provide valuable insights into the decision-making processes and considerations of authors when choosing a publishing avenue. Furthermore, future research could delve deeper into understanding the impact of different research disciplines and the role of subject-specific factors in the citation patterns of hybrid and gold OA publications.

## Conclusion

In conclusion, the findings of this study have noteworthy implications for both researchers and publishers by providing empirical evidence on the impact of hybrid and gold OA journals. The findings suggest that journals’ impact and number of citations did not significantly differ between hybrid or gold OA publishing models. This challenges the idea that publishing in gold OA journals leads to a higher impact as measured by citation metrics such as the IF and CiteScore. The study also emphasizes the need for gold OA journals to offer lower fees and waivers for unfunded authors to promote free access to scientific information. This research sets the stage for further investigations into the various dimensions of open access publishing and its implications for scholarly communication.

## Author contributions

AKT: Conceptualization, methodology, data curation, investigation, supervision, project administration, writing – original draft, and writing – review & editing. NIA: Data curation, investigation, writing – original draft, and writing – review & editing. AOT and ASA: Data curation, investigation, and writing – original draft.

## Ethics and consent statement

Not applicable.

## Data Availability

Open Science Framework: Is publishing gold open-access worth it? An assessment of hybrid and gold open-access publishing models of medical journals on their impact. https://doi.org/10.17605/OSF.IO/SXV3E. ^19^ This project contains the following underlying data:
• Open-access vs. Hybrid journals – Supplementary material Data are available under the terms of the Creative Commons Attribution 4.0 International license (CC-BY 4.0).
