# Does gold open-access publishing increase journal’s impact? An assessment of hybrid versus gold open-access publishing models of medical journals on their impact

**DOI:** 10.12688/f1000research.159550.2

**Published:** 2025-04-07

**Authors:** Abrar K. Thabit, Noha I. Ashy, Abdulrahman O. Fallatah, Ali S. Alquzi

**Affiliations:** 1 Pharmacy Practice Department, Faculty of Pharmacy, King Abdulaziz University, Jeddah, Saudi Arabia; 2 Faculty of Pharmacy, King Abdulaziz University, Jeddah, Saudi Arabia

**Keywords:** publishing, hybrid, open-access, publisher, journal, journal impact factor

## Abstract

**Background:**

Hybrid and gold open-access (OA) are common publishing models. The latter requires fees to allow full-text visibility, whereas hybrid journals offer the option to publish gold OA or for free (subscription-based) where users with access can get the full-text. We evaluated the impact of the publishing model and other factors on medical journals’ impact.

**Methods:**

A sample of hybrid and gold OA medical journals indexed in Web of Science (WOS) and Scopus were included. The effect of the publishing model and other factors on journals’ impact factor (IF), CiteScore, quartile, and number of citations was assessed.

**Results:**

402 journals were included, 201 in each group. Hybrid and gold OA journals had a median age of 32 and 21 years, respectively ( *P*<0.001). While gold OA journals had a slightly higher median IF (3.3 vs. 2.9; *P*=0.021), more hybrid journals had Q1 ranking in Scopus (57.7% vs. 43.8%; *P*=0.091). Publishing model, journal’s age, being of an organization/society, and EMBASE indexation didn’t affect IF, CiteScore, number of citations, and WOS quartile ( *P*>0.05). However, gold OA model wasn’t significantly associated with Scopus Q1 ranking (OR, 0.49; 95% CI, 0.25-0.94; *P*=0.032), indicating that hybrid journals were more likely to have such ranking.

**Conclusion:**

These findings indicate that gold OA publishing doesn’t result in higher impact or journals ranking. Therefore, authors can continue to submit their work to high impact hybrid journals if their interest is getting published in reputable prestigious journals regardless of their publishing model. Gold OA journals are encouraged to reduce their fees to facilitate global research access.

## Introduction

Open access (OA) publications allow individuals to read published scientific articles for free, regardless of location, institutional affiliation, or subscription to the journal (or its publisher). ^1^ Currently, OA publishing model includes several subtypes, the most common type is gold OA, where authors retain the copyright to their published article that is made freely available to any user in the online journal. ^2^ Gold OA publishing model requires the payment of a certain fee by the authors. The other types of OA publishing model include diamond OA, which involves OA publishing without a fee for the author and the reader, and green OA, which permits the placement of the authors’ manuscript in an institutional or a disciplinary repository other than the journal publisher’s website and then the article becomes freely available after an embargo period in some cases. ^1^ While the latter approach is thought to be restricted by limited accessibility and outreach, ^2^ one study found that articles published using green OA model have a comparable citation rate to that of articles published in hybrid journals. ^3^ The concept of gold OA publishing was recently adapted within the last 20 years as journals used to be subscription-based only (i.e., access to articles are only allowed to users subscribed to the journal via their institution or a personal subscription while authors publish their work without a fee). ^3^ However, upon the introduction of gold OA concept, it became widespread among publishers as numerus journals became hybrid, meaning that they allow both subscription-based publishing for authors opting to publish for free and gold OA publishing for authors willing to pay the publication fees. OA publishing is becoming more popular as large research funding agencies, such as the National Institutes of Health in the United States and the Bill and Melinda Gates Foundation, require researchers to make their work freely available. ^2, 4^

Access to medical literature is an essential part of healthcare professionals’ job to make decisions regarding disease management and implementation of certain interventions. Instead of attempting to base their decisions simply on the subset of research to which they have access, clinicians can make more informed health decisions when they have entire access to studies relevant to the cases they are treating. ^5^ OA articles are more frequently read, hence promote access to knowledge and new advances in different disciplines. Since the early 1990s, there has been a notable surge in OA publication, with an estimated annual growth in published articles of 30%. ^6^ Furthermore, a number of universities and journal publishers have recently signed Open Access Transformative Agreements (OATAs), which require a greater transition to OA article publication over a predetermined time period, in response to recommendations from the research communities, such as the Efficiency and Standards for Article Charges Initiative. ^7^ However, the prices of such OATAs are currently very high on the level of APCs charged by hybrid journals for gold OA publishing of articles as such APCs are significantly higher than those of fully gold OA journals. ^4, 5^

Despite the advantages of OA publishing, it has a major disadvantage that should be acknowledged. To publish an OA article, journals ask for publishing fees, which are commonly known as article processing charges (APCs). These charges, which sometimes reach thousands of US dollars, may prevent authors with limited institutional or personal funds from publishing openly and may reflect the for-profit nature of this system. ^6– 9^ The unaffordability of APCs may create a barrier to sharing research. ^10^ Such a major issue influenced many researchers to call for free access to research without barriers (i.e., the need for subscription or forcing authors to pay APCs to make their research accessible to all), such via the advent of diamond OA publishing. ^11^

The prevailing view suggests that the advent of OA publishing model enhances the spread of knowledge and potentially increases citation counts for journals; though, this continues to be contested. In fact, the higher the number of databases a journal is indexed in, the higher the visibility, discoverability and citation of its articles. ^9^ Of note, some databases only index OA journals that regardless of their model (gold, diamond, or green), such as the directory of open-access journals and OpenAlex. At the article level, some studies found that OA articles may have a broader reach and receive citations from various disciplines. ^10^ However, the impact of OA on metrics related to citations was not sufficiently evaluated at the journal level. Although some members of the scientific community have argued that journals’ metrics are considered shallow journal-level rather than article-level metrics, academic institutions still put an emphasis on having their academicians and researchers publish in reputable journals with high metrics for hiring, funding, and promotion purposes. ^11, 12^ Therefore, the objective of this study is to evaluate the effect of the gold OA publishing model on several journals’ metrics on Web of Science (WOS) and Scopus by comparing it to hybrid publishing model.

## Methods

### Journals selection

Medical peer-reviewed journals indexed in Science Citation Index Expanded (SCIE) of WOS and Scopus that are fully published in English were eligible for inclusion in the study. As such, open data, preprint platforms, and public repositories were not included as they are not typically indexed in WOS and Scopus. While journals that publish both clinical and basic sciences studies were included, journals that only publish articles in basic sciences, ethics, healthcare systems management, administration, and policy were excluded. Journals were divided into two groups based on their publishing model as either hybrid or gold OA.

### Definitions

• Hybrid journals: Journals that offer subscription-based and gold OA publishing options. Subscription-based publishing means that only users with access to the journal through an institutional or a personal subscription to the journal can get the full-text of the published articles.
• Gold OA journals: Journals that require authors to pay publishing fees that’s commonly known as APC to have their articles fully visible and available for download to anyone immediately upon publishing.
• Impact factor (IF): 2-year IF of 2021-2022. It’s calculated based on the number of citations of all the articles published in the journal within the last two years.
• CiteScore: 3-year CiteScore of 2019-2022. It’s calculated based on the number of citations of all the articles published in the journal within the last three years.
• Number of citations in WOS: The total number of times articles published in the journal were cited since journal’s inception.
• Number of citations in Scopus: The total number of times articles published in the journal were cited between 2019-2022.
• Quartile: The quartile in which the journal is ranked, where Q1 involves the journals that are ranked the top 25% within a certain specialty, Q2 involves the journals that are ranked between 26-50%, Q3 involves the journals that are ranked between 51-75%, and Q4 involves the journals that are ranked between 76-100%. If a journal is ranked in more than one specialty, the average ranking was calculated.

The IF, CiteScore, number of citations, and quartile of each journal were collected from the journals’ records on Journal Citation Reports and Scopus.

### Statistical analysis

Categorical data were compared using Chi-square test and were presented as numbers and percentages, whereas continuous data were compared using Mann-Whitney test and presented using median [interquartile range (IQR)] as they were deemed skewed based on results of Shapiro-Wilk test of normality. Linear regression was conducted to assess the effect of different factors on IF and CiteScore. Multinomial regression was used to evaluate the association of different factors with Q1 ranking in WOS and Scopus. Odds ratios (OR) and 95% confidence intervals (CI) were reported. This was done to control for potential confounders among the journals’ characteristics in each group and as an alternative to matching journals of each group. Fitting of the regression models was assessed using goodness-of-fit test. Omnibus test of significance was used to assess the significance of the regression models. A *P* value < 0.05 was deemed statistically significant. Analyses were performed on SPSS version 24.0 (IBM Corp., Armonk, NY, USA).

## Results

A total of 402 journals included, 201 in each group. Table 1 shows the characteristics of the included journals. Hybrid and gold OA journals had a median [IQR] age of 32 [23–41.5] and 21 [13–28] years, respectively ( *P* < 0.001). Most of the journals were international (39.3% vs. 53.7%; *P* < 0.001). In terms of volumes and issues, hybrid journals publish more frequently per year than gold OA journals (8 vs. 6; *P* = 0.041). However, 93 (46.3%) of the gold OA publish continuously; thus, were not included in the calculation of the median. More than half of the gold OA journals publish both clinical and basic sciences studies (35.3% vs. 53.2%; *P* < 0.001). The median [IQR] cost of publication in gold OA journals was 2,690 [2,000–2,990]. Although a significant difference was observed in terms of IF between the hybrid and the gold OA publishing models (2.90 [2.20–4.15] vs. 3.30 [2.40–4.45]; *P* = 0.021), no statistically significant difference was observed in terms of CiteScore, and quartiles in both WOS and Scopus ( *P* > 0.05 for all comparisons).

**Table 1.** Characteristics of the journals based on their publishing model.

Characteristic	Hybrid (n=201)	Gold Open-access (n=201)	*P* value
Journal age (years)	32 [23–41.5]	21 [13–28]	< 0.001
Publisher ^*^			< 0.001
Springer	29 (14.4)	48 (23.9)	
Elsevier	38 (18.9)	14 (7)	
Wiley	25 (12.4)	19 (9.4)	
Taylor & Francis	23 (11.4)	13 (6.5)	
Sage	11 (5.5)	18 (9)	
Lippincott Williams & Wilkins	17 (8.5)	1 (0.5)	
Cambridge	7 (3.5)	2 (1)	
Other	51 (25.4)	86 (42.7)	
Journal’s country ^*^			< 0.001
International	79 (39.3)	108 (53.7)	
USA	65 (32.3)	31 (15.4)	
UK	16 (8)	12 (6)	
Canada	10 (5)	4 (2)	
Japan	5 (2.5)	5 (2.5)	
Other	26 (12.9)	41 (20.4)	
Specialty ^*^			< 0.001
General medicine	20 (10)	32 (15.9)	
Neurology/Neurosurgery	18 (9)	11 (5.5)	
Oncology	15 (7.4)	14 (7)	
Cardiology	12 (6)	17 (8.4)	
Endocrinology	13 (6.4)	14 (7)	
Other	123 (61.2)	113 (56.2)	
Publication frequency (per year) ^**^	8 [6–12]	6 [4–12]	0.041
Journal published both clinical and basic sciences	71 (35.3)	107 (53.2)	< 0.001
Publication cost ($)	-	2,690 [2,000–2,990]	-
Year journal switched to gold OA (n=124) ^***^	-	2014 [2008–2019]	-
Journal belongs to an organization	109 (54.2)	96 (47.8)	0.195
Impact factor	2.9 [2.2–4.2]	3.3 [2.4–4.5]	0.021
Quartile in WOS			0.163
Q1	59 (29.4)	52 (25.6)	
Q2	59 (29.4)	81 (40.3)	
Q3	56 (27.9)	48 (23.90	
Q4	27 (13.4)	20 (10)	
Number of citations in WOS ^****^	4,918 [2,510–8,648]	3,963 [2,201–8,915]	0.394
CiteScore	5.10 (3.60–6.85)	5 (3.55–7.10)	0.951
Quartile in Scopus			
Q1	116 (57.7)	88 (43.8)	
Q2	64 (31.8)	83 (41.3)	
Q3	21 (10.4)	30 (14.9)	
Number of citations in Scopus ^****^	2,420 [1,325–4,521]	2,972 [1,307–6,486]	0.060
Indexed in EMBASE	188 (93.5)	181 (90)	0.203

Data are presented as median [interquartile range] or n (%)

OA, open-access; WOS, Web of Science.

^*^ Only top 5 were listed.

^**^ n=93 of gold open-access journals publish continuously (i.e., without assigning articles to a volume or issue); hence, not included in this median.

^***^ The remaining 77 journals were open-access since inception.

^****^ It should be noted that the number of citations doesn’t reflect the number of articles published in a journal.

When different factors were assessed for their effect on IF, only publishing both clinical and basic science studies was significantly associated with higher IF (OR, 2.03; 95% CI,1.19–3.45; *P* = 0.010). The same factor was associated with higher CiteScore (OR,3.63; 95% CI, 1.43–9.20; *P* = 0.007). On the other hand, the publishing model (hybrid vs. gold OA) did not influence IF nor CiteScore (OR, 1.38; 95% CI, 0.81–2.36; *P* = 0.237 and OR,0.81; 95% CI, 0.32–2.04; *P* =0.648, respectively). The effect of different factors on IF and CiteScore are shown in Tables 2 and 3, respectively.

**Table 2.** Effect of different factors on impact factor.

Factor	β Coefficient ± SE	OR (95% confidence interval)	*P* value
Publishing model			0.234
Hybrid	Ref	Ref	
Gold open-access	0.32 ± 0.27	1.38 (0.81–2.36)	
Journal’s age	-0.001 ± 0.007	1 (0.99–1.01)	0.922
Journal published both clinical and basic sciences	0.71 ± 0.27		0.010
Journal belongs to an organization	0.14 ± 0.26	1.15 (0.69–1.91)	0.597
Indexed in EMBASE	0.62 ± 0.48	1.86 (0.72–4.81)	0.200

**Table 3.** Effect of different factors on CiteScore.

Factor	β Coefficient ± SE	OR (95% confidence interval)	*P* value
Publishing model			0. 648
Hybrid	Ref	Ref	
Gold open-access	-0.22 ± 0.47	0.81 (0.32–2.04)	
Journal published both clinical and basic sciences	1.29 ± 0.47	3.63 (1.43–9.2)	0.007
Journal belongs to an organization	0.05 ± 0.46	1.05 (0.43–2.56)	0.921
Indexation in EMBASE	0.79 ± 0.84	2.21 (0.42–11.56)	0.347
Journal’s age	0 ± 0.01	1 (0.98–1.02)	0.982

The effect of different factors on the quartiles of the journals in WOS was evaluated, where none of the factors was associated with ranking in either quartile. Table 4 lists the factors and their effect on ranking in Q1 of WOS. On the other hand, hybrid journals were associated with ranking in Q1 of Scopus as the odds of gold OA journals being ranked as Q1 was less than one (OR, 0.49; 95% CI 0.25–0.94; *P* = 0.032). Table 5 lists the factors and their effect on ranking in Q1 of Scopus.

**Table 4.** Effect of different factors on Q1 ranking in Web of Science.

Factor	OR (95% confidence interval)	*P* value
Publishing model		
Hybrid	Ref	
Gold open-access	1.19 (0.58–2.43)	0.638
Journal published both clinical and basic sciences	1.06 (0.52–2.16)	0.870
Journal belongs to an organization	1.29 (0.65–2.58)	0.468
Indexation in EMBASE	0.38 (0.04–3.33)	0.384
Journal’s age	1.00 (0.99–1.02)	0.914

**Table 5.** Effect of different factors on Q1 ranking in Scopus.

Factor	OR (95% confidence interval)	*P* value
Publishing model		
Hybrid	Ref	
Gold open-access	0.49 (0.25–0.94)	0.032
Journal published both clinical and basic sciences	0.69 (0.36–1.31)	0.257
Journal belongs to an organization	1.50 (0.79–2.83)	0.214
Indexation in EMBASE	< 0.0001 (< 0.0001–< 0.0001)	< 0.0001
Journal’s age	0.99 (0.97–1.00)	0.079

A subgroup analysis in the gold OA group to assess the effect of publishing cost on ranking in WOS showed that higher APCs were associated with Q3 ranking (OR, 2.76; 95% CI, 1.22–6.23; *P* = 0.015) and negatively associated with Q1 ranking (OR, 0.57; 95% CI, 0.26–1.23; *P* < 0.001), but no effect was observed with Q2 ranking. The same observation was found with Q1 ranking in Scopus, where journals with high APCs were less likely to be ranked in Q1 (OR, 0.25; 95% CI, 0.11–0.54; *P* < 0.001).

## Discussion

To our knowledge, this is the first study to evaluate the impact of gold OA publishing model on journals’ metrics. Findings from this study suggest that gold OA publishing model does not have an impact on IF or CiteScore despite the significant difference in IF, that was numerically small but not CiteScore, between hybrid and gold OA journals. These results align with findings from previous studies that explored similar themes, indicating that while OA models aim to increase accessibility, they do not necessarily confer a citation advantage for journals over hybrid models. ^12, 13^ For instance, some studies also found minimal differences in citation metrics between different publishing models, suggesting that other factors, such as journal reputation and article quality, might play more pivotal roles in influencing these metrics. ^14– 16^ Moreover, Piwowar, et al found a significantly higher citation rate of articles published in hybrid journals compared with those published in gild OA journals; though, this could be due to the noticeable difference in the number of articles published subscription-based versus those published gold OA. ^3^ A study by Chua et al found a higher number of citations of OA journals that did not correlate with IF (Spearman’s rho = 0.187; *P* = 0.60). ^16^ Similarly, the increment increase in IF with increased citations was minimal and not statistically significant (β coefficient = 3.35; 95% CI -0.464–7.156; *P* = 0.084). ^16^ As such, it is argued that the very high fees required by gold OA journals (reaching a median [IQR] of $2,690 [2,000–2,990] and a range of $75–5,460) are not justified when considering that many researchers call that science should be free to access by everyone. A study by Butler, et al that compared the financial impact of gold OA publishing by five of the biggest academic publishers estimated that authors paid a total of $1.06 billion in APCs with revenues from gold OA reaching $612.5 million. ^8^ Therefore, such publishers should consider lowering fees to cover necessary editorial processing and offer waivers for unfunded authors provided they provide proof of lack of funds regardless of their country of origin (as many gold OA journals already offer waivers or discounts for authors from low-income countries). This approach aligns with the ethical imperative to democratize access to scientific knowledge, a sentiment echoed in the literature. ^14– 16^

Although our study found that gold OA model wasn’t associated with higher impact in terms of journal metrics, it should be noted that these results don’t reflect the impact of individual articles. ^13, 14^ Overall, journals that publish using only gold OA model market themselves to researchers looking to gain more citations and broader readership. ^15^ Previous research has shown that OA articles, whether they were published using gold OA, green OA, or diamond OA, had higher rates of citations from a broader, diverse readership. A study by Huang, et al found high level of diversity of citing institutions, countries, and fields of research of articles published using OA model. ^13^ Another study by Youg, et al found that interdisciplinary citations were more common with OA articles than those published as non-OA (56% vs. 41%). ^14^ Nevertheless, and from the perspective of the journal level, the current study found no significant difference in the distribution of hybrid and gold OA journals among the different WOS quartiles despite a slightly higher median IF of gold OA journals. This outcome suggests that the prestige and perceived impact and reputation of hybrid journals may still hold sway in the academic community. ^16, 17^ Moreover, in their studies, Gargouri, et al and McCabe, et al have emphasized the importance of publishing both basic science and clinical research to reach a broader audience. The ability of journals that encompass a wide range of disciplines to appeal to a diverse readership has been highlighted as a significant factor in increasing a journal's impact. ^16, 17^ In the current study, this notion was supported by the observation that hybrid journals, which often publish a mix of basic and clinical research, were more likely to achieve higher quartile rankings in Scopus.

Gold OA publishing model is a relatively new concept in research publishing as seen with the significant difference in the median [IQR] ages of hybrid and gold OA journals (32 [23–41.5] vs. 21 [13–28]; *P* < 0.001). Nonetheless, it become widespread, especially as many journals that were formerly hybrid have transitioned to gold OA, where the median [IQR] year of switching was 2014 [2008–2019]. This transition is indicative of the broader shift in the publishing landscape towards more open access models, a trend noted in earlier studies. ^14, 15^ Despite the significant difference in age, it is noteworthy that gold OA journals reached a median total number of citations in WOS since journals’ inception that was not statistically significant from that of hybrid journals, though it was numerically lower (3,963 vs. 4,918; *P* = 0.351). This could be possibly attributed to the large number of gold OA journals (n=93 of 201; 46.3%) that publish articles continuously without assigning them to a certain volume or issue, unlike hybrid journals that have limited quotas. This observation resonates with previous reports that showed that continuous publication models might influence citation patterns differently compared to traditional issue-based models. ^14, 15^

These results of the current study suggest that unfunded researchers who cannot afford gold OA publishing should be less concerned about getting published in subscription-based journals since they can still achieve high impact and rankings. It is worth noting that while articles published in subscription-based hybrid journals may not be freely accessible, readers can still obtain the full text through institutional subscription, by paying for an individual article, or through contacting the corresponding author via email or researchers platforms like ResearchGate and Academia.edu. ^18^ This highlights the role of author accessibility in ensuring the wider dissemination of research findings, regardless of the publishing model. Moreover, hybrid journals that offer gold OA publishing tend to have APCs that are higher than APCs of journals that are fully gold OA by an average of $1,620. ^5^ Nevertheless, the current study highlights the importance of considering the call for free access to scientific information. at minimal costs. Due to these financial barriers (having the authors paying APCs to get their work published through gold OA model or having to pay for a subscription or for an individual article to fully access an article published through a subscription-based model), diamond OA model has emerged allowing for free publishing and free accessibility with no costs to the authors nor to the readers or their institutions. ^2^ While more than 25,000 journals from different disciplines (including approximately 9,000 in science, technologies, engineering, and mathematics that also include medical journals) adopt diamond OA publishing model, the number remains lower than the number of gold OA journals. ^12, 18^ There for andto promote equitable access, gold OA journals and hybrid journals offering gold OA publishing of articles for an APC should offer lower fees and waivers for unfunded authors, regardless of their country of origin. By reducing financial barriers, these journals can facilitate the dissemination of research and contribute to the broader scientific community. Furthermore, hybrid journals should also consider reducing subscription fees, the fees need to be paid by readers to download an individual article, and fees for OATAs.

Of note, the significance of journal metrics vs. article metrics remains controversial where some studies found that the elevated journal metrics are often driven by high citation of only a small number of articles published by a certain journal. ^11^ Despite that, academic institutions, policy makers, and research funders still care about high journal metrics that reflect reputability and prestigiousness for purposes such as funding, incentives, and promotion. Therefore, researchers, such as Suigmoto, et al, has called decision makers to reconsider such focus and shift it to the focus on the quality of the scholarly work and its impact. ^12^

It is important to acknowledge the strengths and limitations of this study. One of the strengths is that it is the first to directly compare the impact of hybrid and gold OA journals at the journal level. By including a large random sample of journals and meeting the target sample size, the study provides a comprehensive analysis of the two models. It also evaluated several factors for their potential association with IF of WOS, CiteScore of Scopus, and quartiles of both databases. However, certain limitations should be considered. The study did not evaluate the impact of gold OA articles published in hybrid journals (also known as paywalled publications), which could potentially influence the overall impact comparison. Unfortunately, such an assessment was not feasible due to the longstanding existence of many hybrid journals. Additionally, journals that offer diamond or green OA, were not included in the current study given their limited number, which could have made the comparison with the abundant hybrid and gold OA unjustified. Lastly, we included a random sample of journals that were not matched based on their characteristics. While the regression analysis that was done in our study may have helped reduce the impact of differences, matching the sample in both groups may improve the interpretation of the results. Future research could explore the impact of these factors on citation metrics and readership. while including a matched set of journals to improve the results. Further research is also warranted to investigate other aspects of OA publishing. While this study focused on IF and CiteScore, future studies could explore additional metrics such as altmetrics, which capture online attention and engagement with scholarly articles. Moreover, qualitative research methods could be employed to understand the perceptions and experiences of researchers regarding hybrid and gold OA publishing models. This would provide valuable insights into the decision-making processes and considerations of authors when choosing a publishing avenue. Furthermore, future research could delve deeper into understanding the impact of different research disciplines and the role of subject-specific factors in the citation patterns of hybrid and gold OA publications.

## Conclusion

In conclusion, the findings of this study have noteworthy implications for both researchers and publishers by providing empirical evidence on the impact of hybrid and gold OA journals. The findings suggest that journals’ impact did not significantly differ between hybrid or gold OA publishing models. This challenges the idea that publishing in gold OA journals leads to a higher impact of journals as measured by citation metrics, such as the IF and CiteScore. The study also emphasizes the need for gold OA journals to offer lower fees and waivers for unfunded authors to promote free access to scientific information. Alternatively, adopting diamond OA model should broaden the accessibility of published research and meet the scientific community’s aspiration of free accessible research. This research sets the stage for further investigations into the various dimensions of open access publishing and its implications for scholarly communication.

## Author contributions

AKT: Conceptualization, methodology, data curation, investigation, supervision, project administration, writing – original draft, and writing – review & editing. NIA: Data curation, investigation, writing – original draft, and writing – review & editing. AOT and ASA: Data curation, investigation, and writing – original draft.

## Ethics and consent statement

Not applicable.

## Data Availability

Open Science Framework: Is publishing gold open-access worth it? An assessment of hybrid and gold open-access publishing models of medical journals on their impact. https://doi.org/10.17605/OSF.IO/SXV3E. ^19^ This project contains the following underlying data:
• Open-access vs. Hybrid journals – Supplementary material Data are available under the terms of the Creative Commons Attribution 4.0 International license (CC-BY 4.0).
