# Association of hybrid versus gold open-access publishing models of medical journals with their impact

**DOI:** 10.12688/f1000research.159550.4

**Published:** 2026-08-04

**Authors:** Abrar K. Thabit, Noha I. Ashy, Abdulrahman O. Fallatah, Ali S. Alquzi

**Affiliations:** 1 Pharmacy Practice Department, Faculty of Pharmacy, King Abdulaziz University, Jeddah, Saudi Arabia; 2 Faculty of Pharmacy, King Abdulaziz University, Jeddah, Saudi Arabia

**Keywords:** publishing, hybrid, open-access, publisher, journal, journal impact factor

## Abstract

**Background:**

Hybrid and gold open-access (OA) are common publishing models. The latter requires fees to allow full-text visibility, whereas hybrid journals offer the option to publish gold OA or for free (subscription-based) where users with access can get the full-text. We evaluated the association of the publishing model and other factors with medical journals’ impact.

**Methods:**

A sample of hybrid and gold OA medical journals indexed in Web of Science (WOS) and Scopus were included. The association of the publishing model and other factors with journals’ impact factor (IF), CiteScore, quartile, and number of citations was assessed.

**Results:**

A total of 402 journals were included, 201 in each group. Hybrid and gold OA journals had a median age of 32 and 21 years, respectively ( *P*<0.001). While gold OA journals had a slightly higher median IF (3.3 vs. 2.9; *P*=0.021), more hybrid journals had Q1 ranking in Scopus (57.7% vs. 43.8%; *P*=0.091). Publishing model, journal’s age, affiliation with organization/society, and EMBASE indexation were not associated with IF, CiteScore, number of citations, and WOS quartile ( *P*>0.05). However, the gold OA model was not significantly associated with the Scopus Q1 ranking (OR, 0.52; 95% CI, 0.34-0.80), indicating that hybrid journals were more likely to have such a ranking.

**Conclusion:**

These findings indicate that gold OA publishing does not result in higher impact or journal ranking. Therefore, authors can continue to submit their work to high impact hybrid journals if their interest is getting published in reputable prestigious journals regardless of their publishing model.

## Introduction

Open access (OA) publications allow individuals to read published scientific articles for free, regardless of location, institutional affiliation, or subscription to the journal (or its publisher). ^1^ Currently, the OA publishing model includes several subtypes. The most common type is gold OA, where authors retain the copyright to their published article, which is made freely available to any user in the online journal. ^2^^,^^3^ The gold OA publishing model requires authors to pay a certain fee. The other types of OA publishing model include diamond OA, which involves OA publishing without a fee for the author and the reader. This type is also named “open access commons” as it allows users to download, read, and distribute the published article at no cost. The third common type of OA is green OA, which permits the authors’ manuscript to be placed in an institutional or a disciplinary repository, or even the author’s own website, other than the journal publisher’s website. In some cases, the article becomes freely available after an embargo period. ^1^ Another variation of green OA is called “post print”, in which the author publishes an online pre-published version of the manuscript that has been accepted after peer review but remains under production by the journal (i.e., not the final version of the article). ^3^ While the latter approach is thought to be restricted by limited accessibility and outreach, ^2^ one study found that articles published using green OA model have a comparable citation rate to that of articles published in hybrid journals. ^4^ The concept of gold OA publishing was recently adapted within the last 20 years as journals used to be subscription-based only (i.e., access to articles is only allowed to users subscribed to the journal via their institution or a personal subscription while authors publish their work without a fee). ^4^ However, upon the introduction of the gold OA concept, it became widespread among publishers as numerus journals became hybrid, meaning that they allow both subscription-based publishing for authors opting to publish for free and gold OA publishing for authors willing to pay publication fees. OA publishing is becoming more popular as large research funding agencies, such as the National Institutes of Health in the United States and the Bill and Melinda Gates Foundation, require researchers to make their work freely available. ^2, 5^

Access to medical literature is an essential part of the job of healthcare professionals to make decisions regarding disease management and implementation of certain interventions. Instead of attempting to base their decisions simply on the subset of research to which they have access, clinicians can make more informed health decisions when they have access to studies relevant to the cases they are treating. ^6^ OA articles are more frequently read, thereby promote access to knowledge and new advances in different disciplines. ^7^ Since the early 1990s, there has been a notable surge in OA publication, with an estimated annual growth of 30% in the number of published articles. ^8^ Furthermore, several universities and journal publishers have recently signed Open Access Transformative Agreements (OATAs), which require a greater transition to OA article publication over a predetermined time period, in response to recommendations from the research communities, such as the Efficiency and Standards for Article Charges Initiative. ^9^ However, the prices of such OATAs are currently very high on the level of APCs charged by hybrid journals for gold OA publishing of articles as such APCs are significantly higher than those of fully gold OA journals. ^5, 6^

Despite the advantages of OA publishing, it has a major drawback that should be acknowledged. To publish an OA article, journals ask for publishing fees, which are commonly known as article processing charges (APCs). These charges, which sometimes reach thousands of US dollars, may prevent authors with limited institutional or personal funds from publishing openly and may reflect the for-profit nature of this system. ^8– 11^ Additionally, papers of a lower quality maybe published in favor of profit-making. Such practice goes against scholarly interests, which may have been associated with the sudden surge in the number of predatory publishers. ^12^ The unaffordability of APCs may create a barrier to research sharing. ^13^ Such a major issue influenced many researchers to call for free access to research without barriers (i.e., the need for subscription or forcing authors to pay APCs to make their research accessible to all), such as the advent of diamond OA publishing. ^14^

The prevailing view suggests that the advent of the OA publishing model enhances the spread of knowledge and potentially increases citation counts for journals; though, this continues to be contested. In fact, the higher the number of databases in which a journal is indexed, the higher the visibility, discoverability and citation of its articles. ^11^ Of note, some databases, such as the directory of open-access journals and OpenAlex, only index OA journals regardless of their model (gold, diamond, or green). At the article level, some studies have found that OA articles may have a broader reach and receive citations from various disciplines. ^13^ However, the association of OA with citation metrics was not sufficiently evaluated at the journal level. Although some members of the scientific community have argued that journals’ metrics are considered shallow journal-level metrics rather than article-level metrics, academic institutions still put an emphasis on having their academicians and researchers publish in reputable journals with high metrics for hiring, funding, and promotion purposes. ^14, 15^ Therefore, the objective of this study is to compare the gold OA publishing model (being the most common OA type) to the hybrid publishing model in terms of journals’ metrics on Web of Science (WOS) and Scopus.

## Methods

### Journals selection

Medical peer-reviewed journals indexed in Science Citation Index Expanded (SCIE) of WOS and Scopus that are fully published in English were eligible for inclusion in the study. As such, open data, preprint platforms, and public repositories were not included as they are not typically indexed in WOS and Scopus. WOS and Scopus were selected as they are major databases with consistent metadata standards, such as citation tracking and peer review verification. Although the database OpenAlex is a growing open source of scholarly metadata, it was not selected because it was reported to to have unique data disambiguation, copyright, and quality issues that currently warrant caution when using it for scientometric studies. ^16^ While journals that publish both clinical and basic sciences studies were included, journals that only publish articles in basic sciences, ethics, healthcare systems management, administration, and policy were excluded. In addition, hybrid journals that later transitioned to gold OA were excluded. Journals were divided into two groups based on their publishing model stated on their website as either hybrid or gold OA.

### Data collection

The list of journals was obtained from the Journal Citation Report (JCR) website of WOS. The list was generated in September 2023. Each journal was screened against the journal selection criteria prior to data collection. The following data were collected individual journal’s websites or their pages on JCR: journal name, publisher, whether it is solely clinical or mixed clinical and basic science, hybrid or gold OA, APC (for gold OA journals only), journal’s country of origin (a journal was deemed international if it has the word “international” in its title, published by an international organization/society/association, or published by a global publisher, such as BioMed Central), specialty, organization or society affiliation, year founded (to calculate the age), number of issues per year (left blank for journals that publish continuously), and indexation in EMBASE database. APCs were converted to US dollars based on the rate on the day the cost data were collected. Journal metrics were collected from JCR of WOS (impact factor and quartile ranking) and Scopus (CiteScore and quartile ranking) databases. Data were collected between November 2023 and February 2024.

### Definitions

• Hybrid journals: Journals that offer subscription-based and gold OA publishing options. Subscription-based publishing means that only users with access to the journal through an institutional or a personal subscription to the journal can get the full-text of the published articles.
• Gold OA journals: Journals that require authors to pay publishing fees, commonly known as APC, to have their articles fully visible and available for download to anyone immediately upon publishing.
• OA articles within hybrid journals: Fully visible articles published in hybrid journals after authors paid APC.
• Non-OA articles within hybrid journals: Articles published in hybrid journals requiring subscription to the journal or payment by the reader to access the full text.
• Fully subscription-based journals: Journals that only publish articles not visible to readers without a subscription to the journal (or publisher) or payment to access an individual article.
• Impact factor (IF): 2-year IF of 2021-2022. It’s calculated based on the number of citations of all the articles published in the journal within the last two years.
• CiteScore: 3-year CiteScore of 2019-2022. It’s calculated based on the number of citations of all the articles published in the journal within the last three years.
• Number of citations in WOS: The total number of times articles published in the journal were cited since the journal’s inception.
• Number of citations in Scopus: The total number of times articles published in the journal were cited between 2019-2022.
• Quartile: The quartile in which the journal is ranked, where Q1 involves the journals that are ranked the top 25% within a certain specialty, Q2 involves the journals that are ranked between 26-50%, Q3 involves the journals that are ranked between 51-75%, and Q4 involves the journals that are ranked between 76-100%. If a journal is ranked in more than one specialty, the average ranking was calculated.
• EMBASE indexing: Journals in the field of biomedicine and pharmacology indexed in EMBASE database. The database currently indexes 8,597 journals. ^17^

The IF, CiteScore, number of citations, and quartile of each journal were collected from the journals’ records on Journal Citation Reports and Scopus.

### Statistical analysis

Categorical data were compared using Chi-square test and presented as numbers and percentages, whereas continuous data were compared using Mann-Whitney test and presented as median [interquartile range (IQR)] as they were deemed skewed based on results of Shapiro-Wilk test of normality. A multivariable generalized linear model (normal distribution with identity link) was conducted to assess the association of different factors with IF and CiteScore. Binary logistic regression was used to evaluate the association of different factors with Q1 ranking vs. non-Q1 ranking, where journals with Q2, Q3, and Q4 rankings were grouped together, in WOS and Scopus, and odds ratios (OR) and 95% confidence intervals (CI) were reported. Hosmer-Lemeshow test was conducted to ensure the goodness of fit of the binary logistic model. These analyses were performed to control for potential confounders among the journals’ characteristics in each group and as an alternative to matching journals of each group. A *P* value < 0.05 was deemed statistically significant. Analyses were performed on SPSS version 24.0 (IBM Corp., Armonk, NY, USA).

## Results

A total of 402 journals were included, with 201 in each group (the dataset is available in the supplementary material). Table 1 shows the characteristics of the included journals. Hybrid and gold OA journals had a median [IQR] age of 32 [23–41.5] and 21 [13–28] years from the year of their inception, respectively ( *P* < 0.001). Most of the journals of both publishing models were international (39.3% vs. 53.7%; *P* < 0.001). In terms of volumes and issues, hybrid journals publish more frequently per year than gold OA journals (8 vs. 6; *P* = 0.041). However, 93 (46.3%) of the gold OA journals publish continuously; thus, were not included in the calculation of the median. More than half of the gold OA journals publish both clinical and basic sciences studies (35.3% vs. 53.2%; *P* < 0.001). The median [IQR] cost of publication in gold OA journals was 2,690 [2,000–2,990]. Although a significant difference was observed in terms of IF between the hybrid and the gold OA publishing models (2.90 [2.20–4.15] vs. 3.30 [2.40–4.45]; *P* = 0.021), no statistically significant difference was observed in terms of CiteScore and quartiles in both WOS and Scopus ( *P* > 0.05 for all comparisons).

**Table 1.** Characteristics of the journals based on their publishing model.

Characteristic	Hybrid (n=201)	Gold Open-access (n=201)	*P* value
Journal age (years)	32 [23–41.5]	21 [13–28]	< 0.001
Publisher ^*^			< 0.001
Springer	29 (14.4)	48 (23.9)	
Elsevier	38 (18.9)	14 (7)	
Wiley	25 (12.4)	19 (9.4)	
Taylor & Francis	23 (11.4)	13 (6.5)	
Sage	11 (5.5)	18 (9)	
Lippincott Williams & Wilkins	17 (8.5)	1 (0.5)	
Cambridge	7 (3.5)	2 (1)	
Other	51 (25.4)	86 (42.7)	
Journal’s country ^*^			< 0.001
International	79 (39.3)	108 (53.7)	
USA	65 (32.3)	31 (15.4)	
UK	16 (8)	12 (6)	
Canada	10 (5)	4 (2)	
Japan	5 (2.5)	5 (2.5)	
Other	26 (12.9)	41 (20.4)	
Specialty ^*^			< 0.001
General medicine	20 (10)	32 (15.9)	
Neurology/Neurosurgery	18 (9)	11 (5.5)	
Oncology	15 (7.4)	14 (7)	
Cardiology	12 (6)	17 (8.4)	
Endocrinology	13 (6.4)	14 (7)	
Other	123 (61.2)	113 (56.2)	
Publication frequency (per year) ^**^	8 [6–12]	6 [4–12]	0.041
Journal published both clinical and basic sciences	71 (35.3)	107 (53.2)	< 0.001
Publication cost ($)	-	2,690 [2,000–2,990]	-
Year journal switched to gold OA (n=124) ^***^	-	2014 [2008–2019]	-
Organization/society affiliation	109 (54.2)	96 (47.8)	0.195
Impact factor	2.9 [2.2–4.2]	3.3 [2.4–4.5]	0.021
Quartile in WOS			0.163
Q1	59 (29.4)	52 (25.6)	
Q2	59 (29.4)	81 (40.3)	
Q3	56 (27.9)	48 (23.90	
Q4	27 (13.4)	20 (10)	
Number of citations in WOS ^****^	4,918 [2,510–8,648]	3,963 [2,201–8,915]	0.394
CiteScore	5.10 (3.60–6.85)	5 (3.55–7.10)	0.951
Quartile in Scopus			0.019
Q1	116 (57.7)	88 (43.8)	
Q2	64 (31.8)	83 (41.3)	
Q3	21 (10.4)	30 (14.9)	
Q4	0 (0)	0 (0)	
Number of citations in Scopus ^****^	2,420 [1,325–4,521]	2,972 [1,307–6,486]	0.060
Indexed in EMBASE	188 (93.5)	181 (90)	0.203

Data are presented as median [interquartile range] or n (%)

OA, open-access; WOS, Web of Science.

^*^ Only top 5 were listed.

^**^ n=93 of gold open-access journals publish continuously (i.e., without assigning articles to a volume or issue); hence, not included in this median.

^***^ The remaining 77 journals were open-access since inception.

^****^ It should be noted that the number of citations doesn’t reflect the number of articles published in a journal.

When different factors were assessed for their association with IF, only publishing both clinical and basic science studies was significantly associated with higher IF (β, 0.71; 95% CI, 0.17–1.24). The same factor was associated with higher CiteScore (β, 1.29; 95% CI, 0.36–2.22). Converesly, the publishing model (hybrid vs. gold OA) did not influence IF nor CiteScore (β, 0.32; 95% CI, -0.21–0.86 and β, -0.22; 95% CI, -1.15–0.71, respectively). The association of different factors with IF and CiteScore are shown in Tables 2 and 3, respectively.

**Table 2.** Association of different factors with impact factor.

Factor	β Coefficient (95% confidence interval)	*P* value
Publishing model		0.234
Hybrid	Ref	
Gold open-access	0.32(-0.21–0.86)	
Journal published both clinical and basic sciences	0.71(0.17–1.24)	0.010
Journal belongs to an organization	0.14(-0.38–0.65)	0.597
Indexed in EMBASE	0.62(-0.33–1.57)	0.200
Journal’s age	-0.001(-0.01–0.12)	0.922

**Table 3.** Association of different factors with CiteScore.

Factor	β Coefficient (95% confidence interval)	*P* value
Publishing model		0. 648
Hybrid	Ref	
Gold open-access	-0.22(-1.15–0.71)	
Journal published both clinical and basic sciences	1.29(0.36–2.22)	0.007
Journal belongs to an organization	0.05(-0.85–0.94)	0.921
Indexation in EMBASE	0.79(-0.86–2.45)	0.347
Journal’s age	0.0001 (-0.02–0.02)	0.982

The association of different factors with the quartiles of the journals in WOS was evaluated, where none of the factors was associated with ranking in either quartile. Table 4 lists the factors and their association with ranking in Q1 of WOS. In contrast, gold OA journals were less associated with ranking in Q1 of Scopus as the odds ratio of was less than one (OR, 0.52; 95% CI 0.34–0.80), which indicates that hybrid journals were more associated with Q1 ranking in Scopus. Table 5 lists the factors and their association with ranking in Q1 of Scopus.

**Table 4.** Association of different factors with Q1 ranking in Web of Science.

Factor	OR (95% confidence interval)	*P* value
Publishing model		
Hybrid	Ref	
Gold open-access	0.924 (0.58–1.46)	0.736
Journal published both clinical and basic sciences	0.943 (0.60–1.50)	0.804
Journal belongs to an organization	1.56 (0.99–2.45)	0.051
Indexation in EMBASE	1.83 (0.72–4.66)	0.205
Journal’s age	1.00 (0.99–1.02)	0.426

**Table 5.** Association of different factors with Q1 ranking in Scopus.

Factor	OR (95% confidence interval)	*P* value
Publishing model		
Hybrid	Ref	
Gold open-access	0.52 (0.34–0.80)	0.002
Journal published both clinical and basic sciences	1.10 (0.73–1.67)	0.658
Journal belongs to an organization	1.17 (0.78–1.74)	0.444
Indexation in EMBASE	0.66 (0.31–1.40)	0.280
Journal’s age	0.99 (0.98–1.00)	0.232

A subgroup analysis in the gold OA group to assess the association of publishing cost with WOS ranking showed that higher APCs were associated with Q3 ranking (OR, 2.76; 95% CI, 1.22–6.23) and negatively associated with Q1 ranking (OR, 0.57; 95% CI, 0.26–1.23), but no association was observed with Q2 ranking. The same observation was found with Q1 ranking in Scopus, where journals with high APCs were less likely to be ranked in Q1 (OR, 0.25; 95% CI, 0.11–0.54).

## Discussion

This study evaluated the association between the gold OA publishing model and journal metrics. Gold OA model is approximately nine years younger than hybrid journals, indicating that it was a concept that was introduced later among medical journals. International journals were the majority among both publishing models. This observation was expected as many popular journals are published by large global publishers, such as BioMed Central journals that are published by Springer. In addition, significantly more gold OA journals publish in clinical and basic sciences disciplines than hybrid journals that seem to be more focused on publishing clinical work. The expansion of the focus by gold OA may reflect the profit-growing interest of the publishers of these journals to attract a larger number of scholarly submissions.

Findings from this study suggest that the gold OA publishing model is not associated with IF or CiteScore despite the significant difference in IF, which was numerically small, between hybrid and gold OA journals. Despite this difference, no difference was observed in the WOS ranking, which indicates that a journal’s rank within its discipline is not impacted by the publishing model. Moreover, significantly more hybrid journals were ranked as Q1 in Scopus compared with gold OA journals. These results align with findings from previous studies that explored similar themes, indicating that while OA models aim to increase accessibility, they do not necessarily confer a citation advantage for journals over hybrid models. ^15, 18^ For instance, some studies also found minimal differences in citation metrics between different publishing models, suggesting that other factors, such as journal reputation and article quality, might play more pivotal roles in influencing these metrics. ^19– 21^ Moreover, Piwowar, et al found a significantly higher citation rate of articles published in hybrid journals compared with those published in gild OA journals; though, this could be due to the noticeable difference in the number of articles published subscription-based versus those published gold OA. ^2^ A study by Chua et al found a higher median number of citations of OA journals compared with non-OA journals (15.5 vs. 12; P = 0.039); though, this did not correlate with IF (Spearman’s rho = 0.187; *P* = 0.60). ^21^ Similarly, the increment increase in IF with increased citations was minimal and not statistically significant (β coefficient = 3.35; 95% CI -0.464–7.156; *P* = 0.084). ^21^ As such, it is argued that the very high fees required by gold OA journals (reaching a median [IQR] of $2,690 [2,000–2,990] and a range of $75–5,460) are not justified when considering that many researchers call for everyone to have free to access to science. A study by Butler, et al that compared the financial impact of gold OA publishing by five of the biggest academic publishers estimated that authors paid a total of $1.06 billion in APCs with revenues from gold OA reaching $612.5 million. ^10^ Therefore, lowering journal APCs aligns with the ethical imperative to democratize access to scientific knowledge, a sentiment echoed in the literature. ^19– 21^

Although our study found that the gold OA model was not associated with higher impact in terms of journal metrics, these results do not reflect the impact of individual articles. ^18, 19^ Overall, journals that only use the gold OA model market themselves to researchers looking to gain more citations and broader readership. ^20^ Previous research has shown that OA articles, whether published using gold OA, green OA, or diamond OA, had higher rates of citations from a broader, diverse readership. A study by Huang, et al found high level of diversity of citing institutions, countries, and fields of research of articles published using OA model. ^22^ Another study by Young, et al found that interdisciplinary citations were more common with OA articles than those published as non-OA (56% vs. 41%). ^23^ Nevertheless, and at the journal level, the current study found no significant difference in the distribution of hybrid and gold OA journals among the different WOS quartiles despite a slightly higher median IF of gold OA journals. This outcome suggests that the prestige and perceived impact and reputation of hybrid journals may still hold sway in the academic community. ^21, 24^ Moreover, in their studies, Gargouri, et al and McCabe, et al have emphasized the importance of publishing both basic science and clinical research to reach a broader audience. The ability of journals that encompass a wide range of disciplines to appeal to a diverse readership has been highlighted as a significant factor in increasing a journal’s impact. ^19, 20^ In the current study, this notion was supported by the observation that hybrid journals, which often publish a mix of basic and clinical research, were more likely to achieve higher quartile rankings in Scopus. Furthermore, the existing literature showed that gold OA publishing has mixed effects on journal impact, with positive results in the fields of Medicine and Life Sciences, whereas the field of Social Sciences and Humanities shows less favorable results. For instance, one study evaluated journals that transitioned from hybrid to gold OA and found a significant increase in relative citation impact after the transition ( *P* < 0.05). ^25^ Similarly, studies by Gumpen-berger, et al and Momeni et al noticed upward trends in various metrics, including IF, SCImago Journal Rank, after conversion to gold OA. ^26^^,^^27^ In contrast, a lack of effect on citation counts with gold OA publishing was observed by Davis et al. (point estimate = 1.01; 95% CI, 0.95–1.07). ^28^ Interestingly, the effect on citation counts may differ based on the field and age of the journal, as observed in a study Björk and Solomon, where journals in Medicine and Life Sciences typically achieve higher impact with gold OA, whereas those in Social Sciences and Humanities tend to score lower. ^15^ The impact on altmetric score was evaluated in a study of 6,000 articles by Chen, et al which found comparable citation counts between articles published OA and those published non-OA. ^29^

Gold OA publishing model is a relatively new concept in research publishing as seen with the significant difference in the median [IQR] ages of hybrid and gold OA journals (32 [23–41.5] vs. 21 [13–28]; *P* < 0.001). Nonetheless, it becomes widespread, especially as many journals that were formerly hybrid have transitioned to gold OA, where the median [IQR] year of switching was 2014 [2008–2019]. This transition is indicative of the broader shift in the publishing landscape towards more open access models, a trend noted in earlier studies. ^19, 20^ Despite the significant difference in age, it is noteworthy that gold OA journals reached a median total number of citations in WOS since journals’ inception that was not statistically significant from that of hybrid journals, though it was numerically lower (3,963 vs. 4,918; *P* = 0.351). This could be possibly attributed to the large number of gold OA journals (n = 93 of 201; 46.3%) that publish articles continuously without assigning them to a certain volume or issue, unlike hybrid journals that have limited quotas. This observation resonates with previous reports that showed that continuous publication models might influence citation patterns differently compared to traditional issue-based models. ^19, 20^

It is worth noting that while articles published in subscription-based hybrid journals may not be freely accessible, readers can still obtain the full text through institutional subscription, by paying for an individual article, or through contacting the corresponding author via email or researchers platforms like ResearchGate and Academia.edu; though, some authors may not be responsive or may fear that their work could be circulated without their knowledge after sharing it with a single user. ^30^ This highlights the role of author accessibility in ensuring the wider dissemination of research findings, regardless of the publishing model. Nonetheless, the lack of responses from authors when reached to share their articles and publisher-imposed embargoes remain barriers to ensuring the accessibility of articles not published as OA in hybrid journals. Shadow libraries may reduce the citation advantage of OA articles. ^31^ Moreover, hybrid journals that offer gold OA publishing tend to have higher APCs than journals that are fully gold OA by an average of $1,620. ^6^ Nevertheless, the current study highlights the importance of considering the call for free access to scientific information at minimal costs. Due to these financial barriers (having the authors pay APCs to get their work published through the gold OA model or having to pay for a subscription or for an individual article to fully access an article published through a subscription-based model), the diamond OA model has emerged, allowing for free publishing and accessibility with no costs to the authors, readers, or their institutions. ^2^ Although more than 25,000 journals from different disciplines (including approximately 9,000 in science, technologies, engineering, and mathematics that also include medical journals) adopt the diamond OA publishing model, the number remains lower than the number of gold OA journals. ^15, 30^ Therefore, gold OA journals and hybrid journals offering gold OA publishing of articles for an APC should offer lower fees and waivers for unfunded authors, regardless of their country of origin to promote equitable access. By reducing financial barriers, these journals can facilitate the dissemination of research and contribute to the broader scientific community. Furthermore, hybrid journals should also consider reducing subscription fees, the fees need to be paid by readers to download an individual article, and fees for OATAs. From the publisher’s perspective, it could be argued that gold OA model offers the advantage of accelerating the path of young journals to achieve high citation volume and broad reach as evidenced by the comparable impact metrics with the hybrid model despite the 9-year gap in the median age difference between hybrid and gold OA journals as shown in the present study. This could also explain the trend of transitioning from hybrid to fully gold OA model by some journals.

Notably, the significance of journal metrics vs. article metrics remains controversial, where some studies found that the elevated journal metrics are often driven by high citations of only a small number of articles published by a certain journal. ^14^ In other words, IF and CiteScore are weak predictors of an individual article’s citation count as some articles in high-metric journals may receive a few or no citations, whereas some articles in journals with low metrics may receive a large number of citations. Despite that, some academic institutions, policy makers, and research funders still care about high journal metrics that reflect reputability and prestigiousness for purposes such as funding, incentives, and promotion. In fact, over the last decade, using citation counts as a proxy for article quality or impact has been criticized. Citation metrics are heavily influenced by factors unrelated to quality, including the size of the discipline and citation practices, and show only a weak association with actual paper relevance. Additionally, both IF and CiteScore are vulnerable to artificial inflation. Therefore, researchers, such as Sugimoto et al, have called on decision makers to reconsider such a focus and shift it to a focus on the quality of scholarly work and its impact. ^15^ It is also recommended that research assessment policies should shift toward evaluating the intrinsic quality and individual-level impact of scholarly work. Furthermore, given that APCs in gold OA journals do not confer a distinct prestige or journal-ranking advantage, publishers and policy bodies face an ethical imperative to dismantle these substantial financial barriers. To democratize access to science without burdening researchers, policies must promote equitable OA models, such as expanding support for diamond and green OA, while promoting lower APCs, fee waivers for unfunded authors regardless of geographic origin, reduced subscription and paywall fees, and shorter publisher embargo periods.

Acknowledging the strengths and limitations of this study is important. A strength of this study is that it is one of a few studies to directly compare hybrid and gold OA journals at the journal level. By including a large random sample of journals and meeting the target sample size, the study provides a comprehensive analysis of the two models. It also evaluated several factors for their potential association with IF of WOS, CiteScore of Scopus, and quartiles of both databases. However, certain limitations should be considered. This study did not evaluate the impact of gold OA articles published in hybrid journals (also known as paywalled publications), which could influence the overall impact comparison. Unfortunately, such an assessment was not feasible due to the long-standing existence of many hybrid journals. Additionally, journals that offer diamond or green OA were not included in the current study given their limited number, which could have made the comparison with the abundant hybrid and gold OA unjustified. Moreover, factors, such as editorial policy and internationalization, were not assessed as potential confounding factors. Finally, we included a random sample of journals that were not matched based on their characteristics. While the regression analysis performed in our study may have helped reduce the impact of differences, matching the sample in both groups may improve the interpretation of the results. Future research could explore the impact of these factors on citation metrics and readership while including a matched set of journals to improve the results. Further research is also warranted to investigate other aspects of OA publishing. Although this study focused on IF and CiteScore, future studies could explore additional metrics, such as altmetrics, which capture online attention and engagement with scholarly articles. Moreover, qualitative research methods could be employed to understand the perceptions and experiences of researchers regarding hybrid and gold OA publishing models. This would provide valuable insights into the decision-making processes and considerations of authors when choosing a publishing avenue. Furthermore, future research could delve deeper into understanding the impact of different research disciplines and the role of subject-specific factors in the citation patterns of hybrid and gold OA publications.

## Conclusion

In conclusion, the findings of this study have noteworthy implications for researchers and publishers by providing empirical evidence on the impact of hybrid and gold OA journals. The findings suggest that the impact of journals did not significantly differ between hybrid and gold OA publishing models. This challenges the idea that publishing in gold OA journals leads to a higher impact of journals as measured by citation metrics, such as the IF and CiteScore. However, the lack of assessment of the impact of articles published as OA in hybrid journals may limit the generalizability of this interpretation. Nevertheless, the adoption of diamond OA model should broaden the accessibility of published research and meet the aspiration of the scientific community for free accessible research. This research sets the stage for further investigations into the various dimensions of open OA and its implications for scholarly communication.

## Author contributions

AKT: Conceptualization, methodology, data curation, investigation, supervision, project administration, writing – original draft, and writing – review & editing. NIA: Data curation, investigation, writing – original draft, and writing – review & editing. AOF and ASA: Data curation, investigation, and writing – original draft.

## Ethics and consent statement

Not applicable.

## Data Availability

Open Science Framework: Is publishing gold open-access worth it? An assessment of hybrid and gold open-access publishing models of medical journals on their impact. https://doi.org/10.17605/OSF.IO/SXV3E. ^32^ This project contains the following underlying data:
• Open-access vs. Hybrid journals – Supplementary material Data are available under the terms of the Creative Commons Attribution 4.0 International license (CC-BY 4.0).
