# Supplementary material for: Association of hybrid versus gold open-access publishing models of medical journals with their impact
Source: F1000Res. 2026 Aug 4;13:1502. Originally published 2024 Dec 9. [Version 4] doi: 10.12688/f1000research.159550.4 (PMC13560058; doi:10.12688/f1000research.159550.4)
Supplement: Peer review history

# Reviewer report: Reviewer response for version 4

**DOI:** 10.5256/f1000research.206587.r503042

**Published:** 2026-08-24

**Authors:** Luiz Alberto Pilatti

**Affiliations:** 1 Universidade Tecnológica Federal do Paraná, Curitiba, Brazil

The manuscript has improved substantially, and the authors have addressed a considerable proportion of the concerns raised during the previous rounds of review. In particular, the framing is now more appropriately focused on associations rather than effects, the methodological description has been expanded, the selection of Web of Science and Scopus has been justified, the non-use of OpenAlex has been explained, and the discussion and policy implications have been strengthened.

 I am therefore willing to amend my previous status from Not Approved to Approved with Reservations.

 A small number of issues should still be corrected before I would consider the manuscript fully approved. Most importantly, there are internal inconsistencies in the reporting of the Scopus Q1 results. In the Abstract, the comparison between hybrid and gold OA journals is reported as 57.7% versus 43.8% with P=0.091, whereas Table 1 reports P=0.019 for the Scopus quartile distribution. These values should be checked and reconciled.

 More importantly, the Abstract states that the gold OA model was “not significantly associated” with Scopus Q1 ranking while simultaneously reporting OR=0.52 with a 95% CI of 0.34–0.80. Table 5 reports the same OR and confidence interval with P=0.002. These results indicate a statistically significant association and therefore contradict the wording used in the Abstract. The statistical interpretation should be corrected consistently throughout the manuscript.

 I also recommend refining the conclusion to ensure that it remains fully consistent with the observational design and the reported results. The statement that gold OA publishing “does not result in higher impact or journal ranking” implies a causal conclusion that cannot be established by this study and does not adequately reflect the significant association observed for Scopus Q1 ranking. A formulation stating that the gold OA model “was not associated with higher IF or CiteScore, while hybrid journals showed higher odds of Q1 ranking in Scopus” would more accurately represent the evidence.

 Similarly, the main conclusion should distinguish the unadjusted comparisons from the adjusted regression results. The manuscript reports a statistically significant unadjusted difference in impact factor and a significant difference in the Scopus quartile distribution, whereas the adjusted analyses do not indicate an association between publishing model and IF or CiteScore. This distinction would make the interpretation more precise.

 These remaining issues are important for internal consistency and accurate interpretation, but they do not, in my assessment, require a fundamental redesign of the study or a new analysis. For this reason, I consider Approved with Reservations the appropriate status for the present version.

Is the work clearly and accurately presented and does it cite the current literature?

Partly

If applicable, is the statistical analysis and its interpretation appropriate?

No

Are all the source data underlying the results available to ensure full reproducibility?

No

Is the study design appropriate and is the work technically sound?

Partly

Are the conclusions drawn adequately supported by the results?

Partly

Are sufficient details of methods and analysis provided to allow replication by others?

Partly

Reviewer Expertise:

NA

I confirm that I have read this submission and believe that I have an appropriate level of expertise to confirm that it is of an acceptable scientific standard, however I have significant reservations, as outlined above.

# Reviewer report: Reviewer response for version 4

**DOI:** 10.5256/f1000research.206587.r507797

**Published:** 2026-08-20

**Authors:** Gergely Ferenc Lendvai

**Affiliations:** 1 Ludovika University of Public Service, Budapest, Hungary

Thank you for the amendments, I do not have further comments on my end as my individual comments have been largely assessed.

 A few important clarifications however, that I think are worth the authors' attention.

 1. "Of note, some databases, such as the directory of open-access journals and OpenAlex, only index OA journals regardless of their model (gold, diamond, or green)." - this is factually incorrect, OAlex indexes non-OA journals as well. And, as a matter of fact, it is reliable in differentiating different OA types as well. Therefore, I think this sentence should be revised. Also, I think it would help to exemplify what the authors meant by "directory of open-access journals", I suppose it is DOAJ but in that case please capitalize it (also its correct name is Directory of Open Access [no -] Journals if I am not mistaken.

 2. Thank you for better outlining what "international" meant. Although there may be room for sophistication (just because the word international appears in a journal title it may not mean that it is indeed international) but I acknowledge that such operationalizations are always subjective, especially in scientometrics. Still, a sentence or two about operationalizing it this way would do good work in the limitations or in the methods.

 I hope that the authors assess the concerns made by my fellow peer reviewers as well, especially those made by Prof. Pilatti more recently but of course R2 and R4 as well. Having read those reviews, I largely agree with them and I opt to not repeat the issues made in those reviews. Thus, I approve this report on the condition that those are assessed as well since my comments have been taken into account. Since there is no option to do indicate this I advise readers to read my review with this caveat in mind.

 Good luck to the authors and thank you for the opportunity to read this paper.

Is the work clearly and accurately presented and does it cite the current literature?

Partly

If applicable, is the statistical analysis and its interpretation appropriate?

Partly

Are all the source data underlying the results available to ensure full reproducibility?

Partly

Is the study design appropriate and is the work technically sound?

Partly

Are the conclusions drawn adequately supported by the results?

Partly

Are sufficient details of methods and analysis provided to allow replication by others?

Partly

Reviewer Expertise:

scientometrics

I confirm that I have read this submission and believe that I have an appropriate level of expertise to confirm that it is of an acceptable scientific standard.

# Reviewer report: Reviewer response for version 3

**DOI:** 10.5256/f1000research.204549.r497842

**Published:** 2026-07-20

**Authors:** Luiz Alberto Pilatti

**Affiliations:** 1 Universidade Tecnológica Federal do Paraná, Curitiba, Brazil

The manuscript has improved substantially. However, the revisions are not yet sufficient for me to amend my previous status because material inconsistencies remain in the statistical reporting and interpretation. 

 The abstract states that the gold OA model was not significantly associated with Scopus Q1 ranking, although the reported estimate is OR = 0.52 with a 95% CI of 0.34–0.80, and Table 5 reports p = 0.002. This is a statistically significant association. The abstract also reports p = 0.091 for the Scopus quartile comparison, whereas the frequencies presented in Table 1 produce a chi-square p value of approximately 0.019. These discrepancies must be corrected consistently throughout the manuscript.

 Important methodological information also remains missing. The manuscript describes the sample as random and states that the target sample size was reached, but it does not explain the sampling procedure, sampling frame, sample-size calculation, or number of eligible journals. The exclusion of hybrid journals that transitioned to gold OA also appears inconsistent with Table 1, which reports 124 journals that transitioned to gold OA.

 The statistical models require further clarification. The authors report that the continuous outcomes were skewed but subsequently apply generalized linear models with a normal distribution and identity link without presenting an adequate justification or model diagnostics. The reference categories, covariate-selection rationale, and results of the stated goodness-of-fit tests should also be reported. In addition, the APC subgroup analysis is not described in the Methods, making it impossible to determine how “higher APCs” was operationalized or how the reported odds ratios were obtained.

 Several interpretations remain inconsistent with the data. The Discussion suggests that hybrid journals more frequently publish both clinical and basic science studies, whereas Table 1 shows a higher proportion among gold OA journals. Gold OA articles published in hybrid journals are also incorrectly described as “paywalled publications.” The conclusion remains too broad because it does not acknowledge the significant adjusted association with Scopus Q1 ranking and continues to employ causal language that is not supported by the observational design.

 The revised version represents meaningful progress, but these problems affect the central findings and their interpretation. Another substantial revision is required before the manuscript can be considered scientifically reliable.

Is the work clearly and accurately presented and does it cite the current literature?

Partly

If applicable, is the statistical analysis and its interpretation appropriate?

No

Are all the source data underlying the results available to ensure full reproducibility?

No

Is the study design appropriate and is the work technically sound?

Partly

Are the conclusions drawn adequately supported by the results?

Partly

Are sufficient details of methods and analysis provided to allow replication by others?

Partly

Reviewer Expertise:

NA

I confirm that I have read this submission and believe that I have an appropriate level of expertise to state that I do not consider it to be of an acceptable scientific standard, for reasons outlined above.

# Reviewer report: Reviewer response for version 3

**DOI:** 10.5256/f1000research.204549.r498836

**Published:** 2026-07-15

**Authors:** Gergely Ferenc Lendvai

**Affiliations:** 1 Ludovika University of Public Service, Budapest, Hungary

Thanks for the opportunity to read this paper. I found it very interesting and I thank the authors for taking the time and energy to write about this important topic. I would also like to thank the authors for taking into account the earlier reviews’ notes – I took a look at them and I found that many concerns have been duly addressed.

 Below are my comments. I truly hope that the authors will find it useful, I made them in the spirit of collegiality and helpfulness rather than “critique”. In order to help the authors, I aimed to follow a fixed structure of my comments so please consult them as follows - I will insert first a passage/part, then make a comment, and then signal whether I find it a minor or major issue.

 1. "The most common type is gold OA, where authors retain the copyright to their published article, which is made freely available to any user in the online journal." - the authors cite a very important paper but it is from 2018. I am not suggesting that the implication does not stand today (although it may be suggested that Green OA is becoming more and more popular, perhaps a quick search on WoS, Scopus or OpenAlex could be of help) but maybe more recent literature could be cited. I am suggesting this so that this sentence does not appear so “categorical” - MINOR

 2. I do think that in the Introduction a much more nuanced understanding and outline are required for the more "naive" reader to better understand the differences between OA types. I anticipate that this paper is mainly aimed at scientometricians and those working on issues related to OA, however, given F1000’s focus and wide-ranging audience it is perhaps important to be a bit more detailed. So, focusing on the Gold is very important but for someone who is not fully familiar with the different types of OA the opening may read a bit complex. I suggest reading the works of Mikael Laakso who has contributed to this field much and his works are excellent in helping outline the structures more. Of course a detailed classification may appear like a stretch but a brief paragraph about the differences would be really helpful and would also help the reasoning behind choosing Gold and Hybrid OA for the analysis – MAJOR. I do see that the authors made an effort to differentiate between some OA types per Prof. Maddi’s comments but if it is not too much to ask for perhaps a bit more detail could be added. I emphasise Prof. Maddi’s earlier comment as well about an issue regarding using Hybrid and Gold as well. I do acknowledge that different OA types may be tricky to distinguish, however, to the best of my knowledge, hybrid journals do partially offer Gold OA and the comparison is a bit difficult because, as Prof. Maddi underlined it rightfully so, these are essentially different models. If you can outline this distinction more, I think your case for the analysis could be more “bulletproof” so to speak.

 3. "OA articles are more frequently read, thereby promote access to knowledge and new advances in different disciplines." - provide a reference please because this is a very categorical sentence and it would add more rigor to the substance. Perhaps you can also cite Craig et al.’s (2007) foundational paper who found that “OA status alone has little or no effect on citations” to add a bit more nuance to the claim. Alternatively, you can also check SciVal although I do think that it would be better to rely on references first as that platform does have a “Views” metadata column where you can check views and may infer to “reads” but this is just a thought you may dismiss it.  - MAJOR

 4. Regarding challenges I think that perhaps more issues could be raised. I am not quite sure if the word "disadvantage" is correct either as it is more of a structural issue rather than a disadvantage. I would focus more on the mechanisms that OA fees trigger – e.g. sometimes publishers are making it mandatory for more profit, and maybe publishing papers of lesser quality because of it. MacLeavy et al. (2020) could be a good guiding source but there are many other papers on this like Frank et al. (2023) or Kaliuzhna et al.’s (2025) more recent scoping review. I am suggesting these to add more meat to the bone as it would make your arguments stronger in my opinion - MINOR

 5. The reasoning behing choosing only SCIE-WoS and Scopus is not entirely clear to me. These databases are often described as having biases and are known for exclusion of many, otherwise excellent journals. May I kindly ask why the authors did not use OpenAlex instead? No need to use it, but as a reader this is a concern that often emerges with similar papers so if you can substantiate your choices that would be great - MAJOR. Also please make sure to give a better reasoning because this argument: “Medical peer-reviewed journals indexed in Science Citation Index Expanded (SCIE) of WOS and Scopus that are fully published in English were eligible for inclusion in the study. As such, open data, preprint platforms, and public repositories were not included as they are not typically indexed in WOS and Scopus” seems rather circular from a reader’s perspective

 6. I suggest providing a summary table of the data in the Methods section for better readability and understanding of the data collection/selection. - MAJOR

 7. "Most of the journals were international (39.3% vs. 53.7%; *P* < 0.001). " - what does international mean in this context? I had a hard time understanding this notion as it could mean a lot of things in scientometrics so operationalizing could help the reader - MINOR

 8. Regarding the Results and the Tables. I found the analysis commendable, however, the descriptions of the results often lack interpretation and remain on the descriptive side. This is not problematic “per se” but some readers’ may find difficult to actually interpret the results so I suggest adding 2-3 sentences to each analytical parts where the authors explain what the results mean in essence. - MAJOR

 9. This is more of a personal note and I can agree with the authors if they provide a good reasoning but when I read the results I could not help but be a bit skeptical about associating factors with quality/prestige markers. Rankings (Q1) or a CiteScore value is composed of many other indicators as well, primarily citation counts, so associating more "peripheral" factors and outlining conclusions thereof may seem a bit like a stretch. I am very much open to hear the authors thoughts on this question.

 10. I have had some difficulties in understanding the logic or rationale in how the discussion points were made. When discussing impact, the authors compare their results to other, larger-scale studies and conclude that "outcome suggests that the prestige and perceived impact and reputation of hybrid journals may still hold sway in the academic community". This is not problematic on its own but maybe it would be better to highlight what may cause the difference between this study and others. This is also a personal note but the current Discussion reads much more like a Lit.Rev. synthesis with little hints of what the study actually came up with as results rather than a “standard” Discussion section in a scientometric paper. Of course, I do not wish to interfere with the authors' logic or understanding of the IMRAD formula and I acknowledge that there are many different schools but perhaps it would be better to contextualise the results of the paper first and maybe add references that are similar or different rather than to list selected studies and underline their findings. It would also help the authors make claims more concretely. I would also encourage the authors to outline policy implications more (based on the results as suggested in the earlier reviews) as OA issues affect virtually every scholarly community even those who have little knowledge about statistical findings. A paragraph about concrete policy implications could be very very helpful in my opinion, or even a table of policy recommendations. - MAJOR

 11. The following paragraph seems a bit fuzzy to me logically: "It is worth noting that while articles published in subscription-based..." First, the authors make a rather optimistic claim that even though some journals are not freely accessible, readers can still find some ways or “tricks” (my wording, not the authors’ but this is how it read to me) to read a paper either via institutional access or by direct messaging authors. But this is the very issue, no? After all, OA should be – in my philosophy – about full-range access. A bigger problem is that RG, Academia edu and all the other sharing services often ignore the actual contractual relation between the publisher and the author and it may cause many issues as well. Furthermore, I would be a bit more cautious and I am not exactly sure whether it's a good alternative to use these platforms to ask for papers as many times the authors do not respond or fear that once they share their paper it may circulate without their knowledge, potentially breaching copyright. This is partially addressed but more nuance should provided. I am not exactly sure either how the conclusion in the second sentence come about since it is a general statement and partially contradicts in substance the first one. A bigger question is about Sci-Hub. Opinions, as always, differ on SH but I would highly advice against promoting, even indirectly as in this case, the use of Sci-Hub as well. Not because it’s “dangerous” but because Sci-Hub is in the gray zone of academic ethics in a very optimistic sense if not outright illegal from a science policy standpoint. I do know that it is a site that is used by lots if not all researchers and of course one can argue that it makes science more accessible, but at the end of the day, it completely disregards the legal issues lying under the publication process and it is also questionable whether it’s ethical that people are uploading your paper to a site for which you may have paid an APC for. Another note is that I also feel like the authors do not fully address the OA question from the publishers' perspective which would be very important and it is a very much under-studied question. Many studies about OA solely cover what could be beneficial for the authors, and what the authors’ challenges are in OA publishing but what about the publishers? Sure, one can make many arguments against the for-profit model but a more nuanced or sophisticated argument could work very well here so that publishers are not portrayed even indirectly as the "bad guys" for enforcing APCs. - MAJOR

 12. I would not use strong phrasings such as the one in the sentence "Despite that, academic institutions, ....". There are many changes happening around the globe, especially today, just think of the new Dutch or Scandinavian models. These statements unfortunately weaken the argument as they stand right now. Or think of the new REF which specifically aims at moving away from metrics. Literature is vast on this, I suggest softening the language a bit and providing more references.

 In sum, thanks again for the opportunity to review this paper and I hope the authors will find my notes to be helpful. Good luck to the authors!

Is the work clearly and accurately presented and does it cite the current literature?

Partly

If applicable, is the statistical analysis and its interpretation appropriate?

Partly

Are all the source data underlying the results available to ensure full reproducibility?

Partly

Is the study design appropriate and is the work technically sound?

Partly

Are the conclusions drawn adequately supported by the results?

Partly

Are sufficient details of methods and analysis provided to allow replication by others?

Partly

Reviewer Expertise:

scientometrics

I confirm that I have read this submission and believe that I have an appropriate level of expertise to confirm that it is of an acceptable scientific standard, however I have significant reservations, as outlined above.

# Response

**Published:** 2026-07-16

**Authors:** Abrar Thabit

**Affiliations:** Pharmacy Practice Department, King Abdulaziz University, Jeddah, Makkah Province, Saudi Arabia

Dear reviewer,

 We would like to thank you for taking the time to review and evaluate our manuscript. We have addressed all your comments and have revised the manuscript accordingly. An updated version of the manuscript highlighting the changes is attached. Please find below the responses to your comments.

 1. "The most common type is gold OA, where authors retain the copyright to their published article, which is made freely available to any user in the online journal." - the authors cite a very important paper but it is from 2018. I am not suggesting that the implication does not stand today (although it may be suggested that Green OA is becoming more and more popular, perhaps a quick search on WoS, Scopus or OpenAlex could be of help) but maybe more recent literature could be cited. I am suggesting this so that this sentence does not appear so “categorical” - MINOR

**Response: **A more recent reference from 2025 (an online article) was added.

 2. I do think that in the Introduction a much more nuanced understanding and outline are required for the more "naive" reader to better understand the differences between OA types. I anticipate that this paper is mainly aimed at scientometricians and those working on issues related to OA, however, given F1000’s focus and wide-ranging audience it is perhaps important to be a bit more detailed. So, focusing on the Gold is very important but for someone who is not fully familiar with the different types of OA the opening may read a bit complex. I suggest reading the works of Mikael Laakso who has contributed to this field much and his works are excellent in helping outline the structures more. Of course a detailed classification may appear like a stretch but a brief paragraph about the differences would be really helpful and would also help the reasoning behind choosing Gold and Hybrid OA for the analysis – MAJOR. I do see that the authors made an effort to differentiate between some OA types per Prof. Maddi’s comments but if it is not too much to ask for perhaps a bit more detail could be added. I emphasise Prof. Maddi’s earlier comment as well about an issue regarding using Hybrid and Gold as well. I do acknowledge that different OA types may be tricky to distinguish, however, to the best of my knowledge, hybrid journals do partially offer Gold OA and the comparison is a bit difficult because, as Prof. Maddi underlined it rightfully so, these are essentially different models. If you can outline this distinction more, I think your case for the analysis could be more “bulletproof” so to speak.

**Response: **Additional details on diamond and green OA were added to the introduction. We also added to the objective statement at the end of the introduction in between parentheses that gold OA is the most common type to justify selecting it for comparison among the other OA types.

 3. "OA articles are more frequently read, thereby promote access to knowledge and new advances in different disciplines." - provide a reference please because this is a very categorical sentence and it would add more rigor to the substance. Perhaps you can also cite Craig et al.’s (2007) foundational paper who found that “OA status alone has little or no effect on citations” to add a bit more nuance to the claim. Alternatively, you can also check SciVal although I do think that it would be better to rely on references first as that platform does have a “Views” metadata column where you can check views and may infer to “reads” but this is just a thought you may dismiss it.  - MAJOR

**Response: **An article was cited for this claim.

 4. Regarding challenges I think that perhaps more issues could be raised. I am not quite sure if the word "disadvantage" is correct either as it is more of a structural issue rather than a disadvantage. I would focus more on the mechanisms that OA fees trigger – e.g. sometimes publishers are making it mandatory for more profit, and maybe publishing papers of lesser quality because of it. MacLeavy et al. (2020) could be a good guiding source but there are many other papers on this like Frank et al. (2023) or Kaliuzhna et al.’s (2025) more recent scoping review. I am suggesting these to add more meat to the bone as it would make your arguments stronger in my opinion - MINOR

**Response: **We replaced the word “disadvantage” with “drawback” to soften the language. We also added an additional sentence supporting this point and cited MacLeavy’s paper.

 5. The reasoning behind choosing only SCIE-WoS and Scopus is not entirely clear to me. These databases are often described as having biases and are known for exclusion of many, otherwise excellent journals. May I kindly ask why the authors did not use OpenAlex instead? No need to use it, but as a reader this is a concern that often emerges with similar papers so if you can substantiate your choices that would be great - MAJOR. Also please make sure to give a better reasoning because this argument: “Medical peer-reviewed journals indexed in Science Citation Index Expanded (SCIE) of WOS and Scopus that are fully published in English were eligible for inclusion in the study. As such, open data, preprint platforms, and public repositories were not included as they are not typically indexed in WOS and Scopus” seems rather circular from a reader’s perspective.

**Response: **Thank you for this comment. We added a justification for using WOS and Scopus and for excluding OpenAlex based on an evidence that was cited (Culbert JH, Hobert A, Jahn N, et al. Reference coverage analysis of OpenAlex compared to Web of Science and Scopus. Scientometrics. 2025;130(4):2475-2492. doi:10.1007/s11192-025-05293-3).

 6. I suggest providing a summary table of the data in the Methods section for better readability and understanding of the data collection/selection. - MAJOR

**Response: **We appreciate this comment. However, the list of the collected data already appear in Table 1 showing the items that were collected and the numerical results. Therefore, no new table was added. Additionally, the full dataset was uploaded and cited in the first sentence of the results section.

 7. "Most of the journals were international (39.3% vs. 53.7%; P < 0.001). " - what does international mean in this context? I had a hard time understanding this notion as it could mean a lot of things in scientometrics so operationalizing could help the reader - MINOR

**Response: **Thank you for this note. We apologize for the ambiguity of this term. We added a clarification in between parentheses on when a journal was deemed international to the “Data collection” subsection of the methods section.

 8. Regarding the Results and the Tables. I found the analysis commendable, however, the descriptions of the results often lack interpretation and remain on the descriptive side. This is not problematic “per se” but some readers’ may find difficult to actually interpret the results so I suggest adding 2-3 sentences to each analytical parts where the authors explain what the results mean in essence. - MAJOR

**Response: **We appreciate the reviewer’s comment. However, interpretation of results and commenting on them are typically reserved for the discussion section. Therefore, and to address this comment, we added to the first paragraph of the discussion a few sentences commenting on the results shown in Table 1.

 9. This is more of a personal note and I can agree with the authors if they provide a good reasoning but when I read the results I could not help but be a bit skeptical about associating factors with quality/prestige markers. Rankings (Q1) or a CiteScore value is composed of many other indicators as well, primarily citation counts, so associating more "peripheral" factors and outlining conclusions thereof may seem a bit like a stretch. I am very much open to hear the authors thoughts on this question.

**Response: **We agree with the reviewer that such metrics can be controversial. Therefore, we already had an argument on this issue in the 6 ^th^ paragraph of the discussion. In the revised version, we expanded this argument based on the comments of reviewer 1 (Lex Bouter).

 10. I have had some difficulties in understanding the logic or rationale in how the discussion points were made. When discussing impact, the authors compare their results to other, larger-scale studies and conclude that "outcome suggests that the prestige and perceived impact and reputation of hybrid journals may still hold sway in the academic community". This is not problematic on its own but maybe it would be better to highlight what may cause the difference between this study and others. This is also a personal note but the current Discussion reads much more like a Lit.Rev. synthesis with little hints of what the study actually came up with as results rather than a “standard” Discussion section in a scientometric paper. Of course, I do not wish to interfere with the authors' logic or understanding of the IMRAD formula and I acknowledge that there are many different schools but perhaps it would be better to contextualise the results of the paper first and maybe add references that are similar or different rather than to list selected studies and underline their findings. It would also help the authors make claims more concretely. I would also encourage the authors to outline policy implications more (based on the results as suggested in the earlier reviews) as OA issues affect virtually every scholarly community even those who have little knowledge about statistical findings. A paragraph about concrete policy implications could be very very helpful in my opinion, or even a table of policy recommendations. - MAJOR

**Response: **We added a sentence to briefly contexualize our result before commenting on them based on previous findings (2 ^nd^ paragraph of the discussion). Regarding policy implications, we expanded the paragraph that hinted on this topic (before the limitations paragraph).

 11. The following paragraph seems a bit fuzzy to me logically: "It is worth noting that while articles published in subscription-based..." First, the authors make a rather optimistic claim that even though some journals are not freely accessible, readers can still find some ways or “tricks” (my wording, not the authors’ but this is how it read to me) to read a paper either via institutional access or by direct messaging authors. But this is the very issue, no? After all, OA should be – in my philosophy – about full-range access. A bigger problem is that RG, Academia edu and all the other sharing services often ignore the actual contractual relation between the publisher and the author and it may cause many issues as well. Furthermore, I would be a bit more cautious and I am not exactly sure whether it's a good alternative to use these platforms to ask for papers as many times the authors do not respond or fear that once they share their paper it may circulate without their knowledge, potentially breaching copyright. This is partially addressed but more nuance should provided. I am not exactly sure either how the conclusion in the second sentence come about since it is a general statement and partially contradicts in substance the first one. A bigger question is about Sci-Hub. Opinions, as always, differ on SH but I would highly advice against promoting, even indirectly as in this case, the use of Sci-Hub as well. Not because it’s “dangerous” but because Sci-Hub is in the gray zone of academic ethics in a very optimistic sense if not outright illegal from a science policy standpoint. I do know that it is a site that is used by lots if not all researchers and of course one can argue that it makes science more accessible, but at the end of the day, it completely disregards the legal issues lying under the publication process and it is also questionable whether it’s ethical that people are uploading your paper to a site for which you may have paid an APC for. Another note is that I also feel like the authors do not fully address the OA question from the publishers' perspective which would be very important and it is a very much under-studied question. Many studies about OA solely cover what could be beneficial for the authors, and what the authors’ challenges are in OA publishing but what about the publishers? Sure, one can make many arguments against the for-profit model but a more nuanced or sophisticated argument could work very well here so that publishers are not portrayed even indirectly as the "bad guys" for enforcing APCs. - MAJOR

**Response: **To address this comment, we added the issues associated with research platforms as suggested. We also removed the example Schi-Hub on shadow libraries (which was initially added based on a comment of a previous reviewer). We added a statement arguing the benefit of gold OA model from the publisher’s perspective to the end of this paragraph.

 12. I would not use strong phrasings such as the one in the sentence "Despite that, academic institutions, ....". There are many changes happening around the globe, especially today, just think of the new Dutch or Scandinavian models. These statements unfortunately weaken the argument as they stand right now. Or think of the new REF which specifically aims at moving away from metrics. Literature is vast on this, I suggest softening the language a bit and providing more references.

**Response: **We agree with the reviewer that not all academic institutions and funders care about these metrics. We added the word “some” to make it less universal. Also, adding the part on policy implications (based comment #10) provides an argument that journal metrics should not be the sole focus when considering research funding, incentives, and promotion, and that a shift of focus should be considered.

 13. In sum, thanks again for the opportunity to review this paper and I hope the authors will find my notes to be helpful. Good luck to the authors!

**Response: **Thank you very much for your valuable review report. We hope that our work has improved after addressing all the comments.

 Respectfully;

 The corresponding author

# Reviewer report: Reviewer response for version 3

**DOI:** 10.5256/f1000research.204549.r497845

**Published:** 2026-07-11

**Authors:** Lex Bouter

**Affiliations:** 1 Vrije Universiteit Amsterdam, Amsterdam, North Holland, The Netherlands

In my opinion the authors responded adequately to my second series of comments. Consequently I upgraded my assessment accordingly.

 However, in my view the authors could have taken a more critical stance with respect to the importance of the Impact Factor (IF) and CiteScore for choosing the journal to which to send a manuscript:

- The IF or CiteScore are at most a weak predictor of the number of citations an article in that journal will get. Also in high IF or CiteScore journals the large majority of articles get no or only a few citations. Furthermore, highly cited articles also occur in low IF of CiteScore journals.
- During the last decade citation counts as a measure for article quality (or impact) has been criticized profoundly. Specifically it has become clear that many other factors, unrelated to article quality (like the magnitude and the citation habits of a disciplinary subfield) have a strong influence on citation counts and also only weak associations between citation count and article quality or relevance have been reported. Furthermore, citation scores and IF can rather easily be inflated artificially.

Is the work clearly and accurately presented and does it cite the current literature?

No

If applicable, is the statistical analysis and its interpretation appropriate?

Partly

Are all the source data underlying the results available to ensure full reproducibility?

No

Is the study design appropriate and is the work technically sound?

No

Are the conclusions drawn adequately supported by the results?

No

Are sufficient details of methods and analysis provided to allow replication by others?

No

Reviewer Expertise:

Research Integrity, Open Science, Methodology

I confirm that I have read this submission and believe that I have an appropriate level of expertise to confirm that it is of an acceptable scientific standard.

# Response

**Published:** 2026-07-16

**Authors:** Abrar Thabit

**Affiliations:** Pharmacy Practice Department, King Abdulaziz University, Jeddah, Makkah Province, Saudi Arabia

Dear reviewer,

 We would like to thank you for taking the time to review and evaluate our manuscript. We have addressed all your comments and have revised the manuscript accordingly. An updated version of the manuscript highlighting the changes is attached. Please find below the responses to your comments.

 1. The IF or CiteScore are at most a weak predictor of the number of citations an article in that journal will get. Also in high IF or CiteScore journals the large majority of articles get no or only a few citations. Furthermore, highly cited articles also occur in low IF of CiteScore journals.

**Response: **We acknowledge this important point, which we already dedicated a paragraph for it in the discussion section (6 ^th^ paragraph). We added this sentence, after rephrasing, for better clarity.

 2. During the last decade citation counts as a measure for article quality (or impact) has been criticized profoundly. Specifically, it has become clear that many other factors, unrelated to article quality (like the magnitude and the citation habits of a disciplinary subfield) have a strong influence on citation counts and also only weak associations between citation count and article quality or relevance have been reported. Furthermore, citation scores and IF can rather easily be inflated artificially.

**Response: **This point is now acknowledged in the 6 ^th^ paragraph of the discussion section.

 Respectfully;

 The corresponding author

# Reviewer report: Reviewer response for version 2

**DOI:** 10.5256/f1000research.179923.r401215

**Published:** 2025-08-20

**Authors:** Abdelghani Maddi

**Affiliations:** 1 CNRS and Sorbonne University, Paris, France

The manuscript examines whether publishing model (Gold OA vs. Hybrid OA) affects journals’ impact, measured by Journal Impact Factor, CiteScore, and quartile rankings in Scopus and Web of Science. The authors conclude that the publishing model does not significantly influence journal impact.

 While the research question is relevant, the study design, data handling, and statistical analyses present major flaws. As a result, the findings are misleading and cannot be validated in their current form. The manuscript also contains misrepresentations of prior literature, questionable policy recommendations, and several methodological omissions.

**I. Major Issues**

**1. Problematic Comparison: Hybrid vs. Gold OA Journals**

 The current design compares Gold OA journals against Hybrid OA journals. However, hybrid journals by definition combine two publishing models: Gold OA and subscription-based. They are not a homogeneous category. Comparing them directly to full Gold OA journals is methodologically unsound, as it conflates two distinct business models within the hybrid group. As a result, the analysis is unable to isolate the effect of open access publishing at the journal level. I strongly recommend to the authors to redefine the study design. At a minimum, distinguish between:

- Gold OA journals,
- OA articles within hybrid journals,
- Non-OA articles within hybrid journals,
- (And ideally, fully subscription-based journals).

Without such separation, the conclusions about “OA vs. hybrid” are invalid in my opinion.

**2. **Incomplete and Biased Treatment of APCs****

 The manuscript discusses article processing charges (APCs) only in the context of Gold OA journals, implicitly portraying them as costly. Yet:

- In practice, APCs in hybrid journals are typically higher than in Gold OA journals, but this important aspect is not acknowledged in the paper. This produces a biased picture of the economics of OA publishing.
- Moreover, APCs are not empirically analyzed in the dataset, so the conclusions about costs are speculative and misplaced.

The authors should, either integrate APC data systematically (for both Gold and Hybrid OA journals) or remove normative claims about costs.

**3. **Lack of Transparency in Data and Methodology****

 Several methodological details are missing or insufficiently explained:

- **Journal model transitions**: The manuscript refers to journal age and model history but does not specify how or from which source the transition dates were determined.
- **EMBASE indexing**: The authors include “indexation in EMBASE” as a variable without explaining what EMBASE is, why it matters, or how it affects citation impact. This appears arbitrary.
- **Regression models:** There are several issues with the statistical analyses that require clarification and correction:
  - **Inappropriate use of odds ratios**: The manuscript reports odds ratios for continuous outcomes such as *Impact Factor* and *CiteScore*. Odds ratios are appropriate for categorical or binary outcomes (e.g., Q1 vs. non-Q1), but not for raw continuous metrics. If the authors transformed or dichotomized these variables, this must be explicitly stated and justified. As currently presented, the results are misleading.
  - **Lack of transparency in model specification**: The regression models are insufficiently described. It is unclear which transformations were applied, what the reference categories are, and how the models were evaluated. Without this information, the analyses are not reproducible.
  - **Inconsistent interpretation of results**: In Table 4, Gold OA is significantly associated with a lower likelihood of being Q1 in Scopus (OR = 0.49, p = 0.032). However, the abstract and discussion state that there is “no significant effect.”.

Taken together, these issues suggest that the statistical analyses are not being applied or reported appropriately. At minimum, the authors must clarify the modeling approach, correct the misuse of odds ratios, and ensure that the interpretation of significant results is consistent throughout the paper.

**4. Problematic and Unsupported Claims**

 The policy recommendations presented in the manuscript are not evidence-based and, in some cases, are contradictory or misleading. For example (not exhaustive list):

- The authors suggest that *“readers can still obtain the full text through institutional subscription, by paying for an individual article, or through contacting the corresponding author via email or researcher platforms like ResearchGate and Academia.edu.”*
  This recommendation ignores key issues: access by payment contradicts the critique of APCs as a barrier, and informal sharing through ResearchGate or Academia.edu disregards copyright restrictions and the fact that legally shareable versions are often limited to preprints or accepted manuscripts (after embargo).
- The claim that *“researchers who cannot afford gold OA publishing should be less concerned about getting published in subscription-based journals since they can still achieve high impact and rankings”* is highly problematic. It disregards equity issues in access to knowledge and implicitly endorses the continuation of paywalled dissemination, which is contrary to the goals of open science.
- Finally, the statement that *“the study emphasizes the need for gold OA journals to offer lower fees and waivers for unfunded authors to promote free access to scientific information”* is not grounded in the presented analysis. APC levels are not included in the data or models, and the comparison ignores the fact that APCs in hybrid journals are generally higher than APCs of fully Gold OA journals.

Taken together, these claims are speculative, not supported by the study’s findings, and risk reinforcing the status quo rather than providing meaningful policy guidance.

 I suggest to remove or thoroughly revise the recommendations. They are normative, inconsistent with the results, and contradict much of the existing OA literature.

**5. Misrepresentation of Prior Literature**

 The manuscript claims to be the first to study the effect of publishing models at the journal level. This is demonstrably false:

 Existing literature showed that Gold OA publishing shows mixed effects on journal impact, with positive results in Medicine and Life Sciences but lower impact in Social Sciences and Humanities. For example (see below the list of related papers I am aware of), Bautista-Puig et al. (2020) report a significant post‐conversion increase in relative citation impact (p < 0.05), and Chua et al. (2017) find that gold OA journals receive 1.3 times more citations (p = 0.039) compared with non–open access journals. Studies by Gumpen- berger et al. (2013) and Momeni et al. (2021) note upward trends in metrics such as Journal Impact Factor, SCImago Journal Rank, and other normalized citation measures after a shift to gold OA, even if precise effect sizes are not given.

 In contrast, Davis (2011) finds no effect on citation counts (point estimate = 1.01; 95% CI, 0.95–1.07), and Björk and Solomon (2012) observe that initial differences in citation rates between open and subscription journals disappear when factors like discipline and age are controlled. Findings differ by field: journals in Medicine and Life Sciences typically achieve higher impact with gold OA, whereas Social Sciences and Humanities tend to score lower.

 The novelty claim must be corrected, and these references properly integrated.

 Moreover, the study ignores the role of Sci-Hub and shadow libraries, which provide access to paywalled articles and reduce the measurable citation advantage of OA (see Maddi et Sapinho 2023). To have more robust results, this effect should be neutralized.

 II. Minor Comments

- Write “Sugimoto” instead of “Suigmoto”.
- The title is misleading: it implies a causal claim (“Does OA increase impact?”) while the design is more observational.
- The abstract misinterprets results (e.g., underreporting the significant association in Scopus quartiles).

To sum up, the paper requires major restructuring: from redefining the comparison groups to improving the statistical models, correcting the literature claims, and removing unsupported recommendations. As it stands, the article is misleading and fails to meet the methodological rigor required for indexing in F1000Research.

Is the work clearly and accurately presented and does it cite the current literature?

Partly

If applicable, is the statistical analysis and its interpretation appropriate?

No

Are all the source data underlying the results available to ensure full reproducibility?

Partly

Is the study design appropriate and is the work technically sound?

No

Are the conclusions drawn adequately supported by the results?

Partly

Are sufficient details of methods and analysis provided to allow replication by others?

Partly

Reviewer Expertise:

Open Science, Scientometrics, Research Policy, Economics

I confirm that I have read this submission and believe that I have an appropriate level of expertise to state that I do not consider it to be of an acceptable scientific standard, for reasons outlined above.

## References

[1] : Do journals flipping to gold open access show an OA citation or publication advantage?. *Scientometrics* .2020;124(3) : 10.1007/s11192-020-03546-x2551-2575 10.1007/s11192-020-03546-x

[2] : Open access versus subscription journals: a comparison of scientific impact. *BMC Medicine* .2012;10(1) : 10.1186/1741-7015-10-73 10.1186/1741-7015-10-73 PMC339885022805105

[3] : The impact factor of an open access journal does not contribute to an article’s citations. *F1000Research* .2017;6: 10.12688/f1000research.10892.1 10.12688/f1000research.10892.1 PMC546422028649365

[4] : Open access, readership, citations: a randomized controlled trial of scientific journal publishing. *The FASEB Journal* .2011;25(7) : 10.1096/fj.11-1839882129-2134 10.1096/fj.11-183988 21450907

[5] : On the impact of Gold Open Access journals. *Scientometrics* .2013;96(1) : 10.1007/s11192-012-0902-7221-238 10.1007/s11192-012-0902-7

[6] : On the culture of open access: the Sci-hub paradox. *Scientometrics* .2023;128(10) : 10.1007/s11192-023-04792-55647-5658 10.1007/s11192-023-04792-5

[7] : What happens when a journal converts to open access? A bibliometric analysis. *Scientometrics* .2021;126(12) : 10.1007/s11192-021-03972-59811-9827 10.1007/s11192-021-03972-5

[8] : The citation performance of open access journals: A disciplinary investigation of citation distribution models. *Journal of the American Society for Information Science and Technology* .2007;58(13) : 10.1002/asi.206762145-2156 10.1002/asi.20676

[9] : Disentangling Gold Open Access. 10.1007/978-3-030-02511-3_5129-144 10.1007/978-3-030-02511-3_5

# Response

**Published:** 2026-06-20

**Authors:** Abrar Thabit

**Affiliations:** Pharmacy Practice Department, King Abdulaziz University, Jeddah, Makkah Province, Saudi Arabia

Dear reviewer,

 We would like to thank you for taking the time to review and evaluate our manuscript. We have addressed all your comments and have revised the manuscript accordingly. An updated version of the manuscript highlighting the changes is attached. Please find below the responses to your comments.

**Major comments:**

**1. Problematic Comparison: Hybrid vs. Gold OA Journals**The current design compares Gold OA journals against Hybrid OA journals. However, hybrid journals by definition combine two publishing models: Gold OA and subscription-based. They are not a homogeneous category. Comparing them directly to full Gold OA journals is methodologically unsound, as it conflates two distinct business models within the hybrid group. As a result, the analysis is unable to isolate the effect of open access publishing at the journal level. I strongly recommend to the authors to redefine the study design. At a minimum, distinguish between:

-
  - Gold OA journals,
  - OA articles within hybrid journals,
  - Non-OA articles within hybrid journals,
  - (And ideally, fully subscription-based journals).

Without such separation, the conclusions about “OA vs. hybrid” are invalid in my opinion.

**Response: **The missing definitions were added to the definitions subsection of the methods section to add further clarification of these distinguished terms.

 2. **Incomplete and Biased Treatment of APCs**

1.
  - The manuscript discusses article processing charges (APCs) only in the context of Gold OA journals, implicitly portraying them as costly. Yet:
  - In practice, APCs in hybrid journals are typically higher than in Gold OA journals, but this important aspect is not acknowledged in the paper. This produces a biased picture of the economics of OA publishing.
  - Moreover, APCs are not empirically analyzed in the dataset, so the conclusions about costs are speculative and misplaced.

The authors should, either integrate APC data systematically (for both Gold and Hybrid OA journals) or remove normative claims about costs.

**Response: **Claims and recommendations to publishers about costs were removed from the discussion section as this was also suggested by reviewer 1.

**3. Lack of Transparency in Data and Methodology **Several methodological details are missing or insufficiently explained:

-
  - **Journal model transitions:** The manuscript refers to journal age and model history but does not specify how or from which source the transition dates were determined.
  - **EMBASE indexing:** The authors include “indexation in EMBASE” as a variable without explaining what EMBASE is, why it matters, or how it affects citation impact. This appears arbitrary.
  - **Regression models:** There are several issues with the statistical analyses that require clarification and correction:
    -
      - **Inappropriate use of odds ratios:** The manuscript reports odds ratios for continuous outcomes such as Impact Factor and CiteScore. Odds ratios are appropriate for categorical or binary outcomes (e.g., Q1 vs. non-Q1), but not for raw continuous metrics. If the authors transformed or dichotomized these variables, this must be explicitly stated and justified. As currently presented, the results are misleading.
      - **Lack of transparency in model specification:** The regression models are insufficiently described. It is unclear which transformations were applied, what the reference categories are, and how the models were evaluated. Without this information, the analyses are not reproducible.
      - **Inconsistent interpretation of results:** In Table 4, Gold OA is significantly associated with a lower likelihood of being Q1 in Scopus (OR = 0.49, p = 0.032). However, the abstract and discussion state that there is “no significant effect.”.

Taken together, these issues suggest that the statistical analyses are not being applied or reported appropriately. At minimum, the authors must clarify the modeling approach, correct the misuse of odds ratios, and ensure that the interpretation of significant results is consistent throughout the paper.

**Response: **Please see below the respective response to each point:

- **Journal model transitions: **In our dataset, we did not include journals that transitioned from hybrid to gold OA to clearly distinguish between journals that have remained hybrid and those that remained gold OA at the time of data collection and analysis. We only mentioned this in the discussion section to clarify the current trend of certain journals. To clarify this issue, we added to the methods section the fact that we excluded journals that transitioned from hybrid to gold OA.
- **EMBASE indexing: **A definition was added to the definitions subsection of the methods section. EMBASE is a known database to researchers in the field of biomedicine. This is why we included it as a variable since our study focuses on medical journals.
- **Regression models: **Thank you for this valuable note. After consulting a statistician to address this comment, we realized certain issues with our analysis. First, we corrected the name of the first analysis (that was reported in tables 2 and 3) from “linear regression” to “multivariable generalized linear model (normal distribution with identity link).” Second, we removed all the OR (95% confidence interval) columns from tables 2 to 5 since these were incorrectly reported. Instead, we kept the β coefficient column and replaced standard error with 95% confidence interval. The reporting in both the results text and the abstract were corrected. Third, for the association of factors with journal quartiles (that were reported in tables 4 and 5), binary logistic regression was done to replace multinomial regression. The terminology was edited in the methods section, and the results were updated in the results text and tables 4 and 5 accordingly. We hope that these major changes ensure the correctness and integrity of the reported results. We also ensured the consistency of reporting the results in both the main text and the abstract.

**4. Problematic and Unsupported Claims**

 The policy recommendations presented in the manuscript are not evidence-based and, in some cases, are contradictory or misleading. For example (not exhaustive list):

-
  - The authors suggest that “readers can still obtain the full text through institutional subscription, by paying for an individual article, or through contacting the corresponding author via email or researcher platforms like ResearchGate and Academia.edu.” This recommendation ignores key issues: access by payment contradicts the critique of APCs as a barrier, and informal sharing through ResearchGate or Academia.edu disregards copyright restrictions and the fact that legally shareable versions are often limited to preprints or accepted manuscripts (after embargo).
  - The claim that “researchers who cannot afford gold OA publishing should be less concerned about getting published in subscription-based journals since they can still achieve high impact and rankings” is highly problematic. It disregards equity issues in access to knowledge and implicitly endorses the continuation of paywalled dissemination, which is contrary to the goals of open science.
  - Finally, the statement that “the study emphasizes the need for gold OA journals to offer lower fees and waivers for unfunded authors to promote free access to scientific information” is not grounded in the presented analysis. APC levels are not included in the data or models, and the comparison ignores the fact that APCs in hybrid journals are generally higher than APCs of fully Gold OA journals.

Taken together, these claims are speculative, not supported by the study’s findings, and risk reinforcing the status quo rather than providing meaningful policy guidance.

 I suggest to remove or thoroughly revise the recommendations. They are normative, inconsistent with the results, and contradict much of the existing OA literature.

**Response: **Regarding the first point, we added a sentence following this one reiterating the difficulty of accessing articles not published as OA in hybrid journals, such as in the case of lack of response from authors when contacted to share their articles, as well as certain embargos imposed by publishers.

 Regarding the second point, the sentence was removed.

 Regarding the third point, this sentence was removed and replaced by the following sentence after mentioning that publishing model wasn’t associated with impact factor or CiteScore “However, lack of assessing the impact of articles published OA in hybrid journals may limit the generalizability of this interpretation.” Similarly, the following sentence was removed from the first paragraph of the discussion based on a suggestion from reviewer 1 “Therefore, such publishers should consider lowering fees to cover necessary editorial processing and offer waivers for unfunded authors provided they provide proof of lack of funds regardless of their country of origin (as many gold OA journals already offer waivers or discounts for authors from low-income countries).”

**5. Misrepresentation of Prior Literature **The manuscript claims to be the first to study the effect of publishing models at the journal level. This is demonstrably false:

 Existing literature showed that Gold OA publishing shows mixed effects on journal impact, with positive results in Medicine and Life Sciences but lower impact in Social Sciences and Humanities. For example (see below the list of related papers I am aware of), Bautista-Puig et al. (2020) report a significant post‐conversion increase in relative citation impact (p < 0.05), and Chua et al. (2017) find that gold OA journals receive 1.3 times more citations (p = 0.039) compared with non–open access journals. Studies by Gumpen- berger et al. (2013) and Momeni et al. (2021) note upward trends in metrics such as Journal Impact Factor, SCImago Journal Rank, and other normalized citation measures after a shift to gold OA, even if precise effect sizes are not given.

 In contrast, Davis (2011) finds no effect on citation counts (point estimate = 1.01; 95% CI, 0.95–1.07), and Björk and Solomon (2012) observe that initial differences in citation rates between open and subscription journals disappear when factors like discipline and age are controlled. Findings differ by field: journals in Medicine and Life Sciences typically achieve higher impact with gold OA, whereas Social Sciences and Humanities tend to score lower.

 The novelty claim must be corrected, and these references properly integrated.

 Moreover, the study ignores the role of Sci-Hub and shadow libraries, which provide access to paywalled articles and reduce the measurable citation advantage of OA (see Maddi et Sapinho 2023). To have more robust results, this effect should be neutralized.

**Response: **We reworded the first sentence of the discussion and removed the word “first.” We also removed it from the last paragraph. We included the studies suggested in the second paragraph of the discussion with a brief description of their findings as stated (The study by Chua et al was already cited and discussed in the first paragraph, but we added the observation stated in this comment to it). We also included a brief sentence on shadow libraries in the discussion section as suggested and we cited the reference mentioned.

**Minor comments**

 1. Write “Sugimoto” instead of “Suigmoto”.

**Response: **Corrected.

 2. The title is misleading: it implies a causal claim (“Does OA increase impact?”) while the design is more observational.

**Response: **The first part of the title was removed. It was reworded to “Association of hybrid versus gold open-access publishing models of medical journals with their impact” to avoid implying casualty.

 3. The abstract misinterprets results (e.g., underreporting the significant association in Scopus quartiles).

**Response: **This part was corrected based on the above comment, as well as a comment from reviewer 1.

 4. To sum up, the paper requires major restructuring: from redefining the comparison groups to improving the statistical models, correcting the literature claims, and removing unsupported recommendations. As it stands, the article is misleading and fails to meet the methodological rigor required for indexing in F1000Research.

**Response: **We appreciate the valuable comments made. We hope that the revised manuscript is an improved version of our work.

 Respectfully;

 The corresponding author

# Reviewer report: Reviewer response for version 2

**DOI:** 10.5256/f1000research.179923.r401219

**Published:** 2025-08-19

**Authors:** Luiz Alberto Pilatti

**Affiliations:** 1 Universidade Tecnológica Federal do Paraná, Curitiba, Brazil

The manuscript investigates whether publishing under the gold open access model is associated with a higher impact of medical journals than the hybrid model. To this end, the authors collected bibliometric indicators such as impact factor, CiteScore, SJR, and SNIP and compared journals with different access models. The study concludes that journals in the gold OA model tend to present higher impact than hybrid journals.

 The work is presented clearly and well-organized, facilitating reading, but the literature review is still limited. The authors do not explore recent references discussing the differences between hybrid and fully open access models, nor do they include debates on alternative metrics such as altmetrics or indicators of digital visibility. Incorporating more recent studies would strengthen the theoretical and contextual basis of the manuscript.

 The study design is relevant but presents weaknesses that compromise its technical soundness. The criteria for selecting the journals are not sufficiently clear, and there is no detail on which subject areas were included or excluded. Furthermore, potential confounding factors such as journal age, editorial policy, or internationalization, which could influence the indicators, were not considered. A more robust methodological clarification and a discussion of these biases are necessary.

 The current methodological description does not allow other researchers to replicate the study. Precise information about the data sources used, the date of collection, the inclusion and exclusion criteria, and how journals were classified as hybrid or gold OA is missing. Without these elements, the study loses transparency and reproducibility. Detailing these aspects is essential to ensure the credibility of the work.

 About statistical analysis, the study presents only descriptive comparisons without applying significance tests to support the observed differences. This absence weakens the conclusions, which are more speculative than scientific. Applying appropriate tests, such as t-test, ANOVA, or multivariate regression, would allow the hypotheses to be validated or refuted with greater robustness. If the authors choose not to include statistical analyses, the conclusions need to be reformulated in more cautious terms.

 Another critical point is the lack of raw data used in the analysis. Without these materials, it is impossible to verify the results' consistency or ensure the study's reproducibility. It is recommended that the authors provide a dataset with the collected indicators, specifying the source and year of each data point, preferably in an open-access repository. This would increase the transparency and scientific utility of the work.

 The conclusions presented are broader than the results can support. Given the absence of statistical analysis and the methodological limitations, it is impossible to state categorically that gold OA journals have a higher impact than hybrid ones. The conclusions should be reformulated to reflect the preliminary and exploratory nature of the findings, acknowledging the study's limitations and avoiding excessive generalizations.

 The article addresses a relevant and timely issue that can contribute to the debate on scientific publishing models, but requires substantial revisions before it can be considered solid. Expanding the literature review, detailing the methodology, including statistical analyses, making the raw data available, and reformulating the conclusions are indispensable steps. If these points are adequately addressed, the work may significantly contribute to discussing the impact of open access models in medical journals.

Is the work clearly and accurately presented and does it cite the current literature?

Partly

If applicable, is the statistical analysis and its interpretation appropriate?

No

Are all the source data underlying the results available to ensure full reproducibility?

No

Is the study design appropriate and is the work technically sound?

Partly

Are the conclusions drawn adequately supported by the results?

Partly

Are sufficient details of methods and analysis provided to allow replication by others?

Partly

Reviewer Expertise:

Health Sciences, Public Health, Bibliometrics, Scientific Communication, Open Access Publishing.

I confirm that I have read this submission and believe that I have an appropriate level of expertise to state that I do not consider it to be of an acceptable scientific standard, for reasons outlined above.

## References

[1] : Open access versus subscription journals: a comparison of scientific impact. *BMC Medicine* .2012;10(1) : 10.1186/1741-7015-10-73 10.1186/1741-7015-10-73 PMC339885022805105

[2] : Hybrid Gold Open Access Citation Advantage in Clinical Medicine: Analysis of Hybrid Journals in the Web of Science. *Publications* .2023;11(2) : 10.3390/publications11020021 10.3390/publications11020021

# Response

**Published:** 2026-06-20

**Authors:** Abrar Thabit

**Affiliations:** Pharmacy Practice Department, King Abdulaziz University, Jeddah, Makkah Province, Saudi Arabia

Dear reviewer,

 We would like to thank you for taking the time to review and evaluate our manuscript. We have addressed all your comments and have revised the manuscript accordingly. An updated version of the manuscript highlighting the changes is attached. Please find below the responses to your comments.

 1. The work is presented clearly and well-organized, facilitating reading, but the literature review is still limited. The authors do not explore recent references discussing the differences between hybrid and fully open access models, nor do they include debates on alternative metrics such as altmetrics or indicators of digital visibility. Incorporating more recent studies would strengthen the theoretical and contextual basis of the manuscript.

**Response: **Additional studies with brief explanation of their findings were added to the end of the second paragraph of the discussion, including a study that evaluated the impact on altmetric score. We also included that the evaluation on altmetrics is warranted in future studies in the last paragraph of the discussion before the conclusion.

 2. The study design is relevant but presents weaknesses that compromise its technical soundness. The criteria for selecting the journals are not sufficiently clear, and there is no detail on which subject areas were included or excluded. Furthermore, potential confounding factors such as journal age, editorial policy, or internationalization, which could influence the indicators, were not considered. A more robust methodological clarification and a discussion of these biases are necessary.

**Response: **We believe that the “Journal selection” subsection of the methods section clearly lists the inclusion and exclusion criteria. We specifically mentioned that we included medical journals published in English and indexed in WOS and Scopus. We also mentioned that we excluded journals of basic sciences, ethics, healthcare systems management, administration, and policy. For further clarification, we added the exclusion criterion of the journals that transitioned from hybrid to gold OA.

 With regards to the factors, we did include journal age as a factor in all the analyses. However, we listed “the lack of assessing of editorial policy and internationalization as potential confounding factor” as a limitation in the last paragraph of the discussion.

 3. The current methodological description does not allow other researchers to replicate the study. Precise information about the data sources used, the date of collection, the inclusion and exclusion criteria, and how journals were classified as hybrid or gold OA is missing. Without these elements, the study loses transparency and reproducibility. Detailing these aspects is essential to ensure the credibility of the work.

**Response: **We added a new subsection to the methods section titled “Data collection”, where we mentioned the data collected, source of the data collected, and the dates the study was conducted. Regarding inclusion and exclusion criteria and how the journals were classified, this was already described under the “Journal selection” subsection of the methods.

 4. About statistical analysis, the study presents only descriptive comparisons without applying significance tests to support the observed differences. This absence weakens the conclusions, which are more speculative than scientific. Applying appropriate tests, such as t-test, ANOVA, or multivariate regression, would allow the hypotheses to be validated or refuted with greater robustness. If the authors choose not to include statistical analyses, the conclusions need to be reformulated in more cautious terms.

**Response: **We employed Chi-square and Mann-Whitney tests for comparison purposes. However, for the assessment of factors and their association with different metrics, we employed multivariable generalized linear model and binary logistic regression. We consulted a statistician who improved our statistics in this revised version of the manuscript after a similar comment was made by reviewer 4. We updated the results accordingly as shown by the marked text.

 5. Another critical point is the lack of raw data used in the analysis. Without these materials, it is impossible to verify the results' consistency or ensure the study's reproducibility. It is recommended that the authors provide a dataset with the collected indicators, specifying the source and year of each data point, preferably in an open-access repository. This would increase the transparency and scientific utility of the work.

**Response: **We indeed uploaded the dataset as a supplementary material. However, we apologize for not citing that in the main text. We have now added that clarification in the first sentence of the results.

 6. The conclusions presented are broader than the results can support. Given the absence of statistical analysis and the methodological limitations, it is impossible to state categorically that gold OA journals have a higher impact than hybrid ones. The conclusions should be reformulated to reflect the preliminary and exploratory nature of the findings, acknowledging the study's limitations and avoiding excessive generalizations.

**Response: **We added the following sentence to the conclusion section to tone down our conclusion “However, lack of assessing the impact of articles published OA in hybrid journals may limit the generalizability of this interpretation.”

 7. The article addresses a relevant and timely issue that can contribute to the debate on scientific publishing models but requires substantial revisions before it can be considered solid. Expanding the literature review, detailing the methodology, including statistical analyses, making the raw data available, and reformulating the conclusions are indispensable steps. If these points are adequately addressed, the work may significantly contribute to discussing the impact of open access models in medical journals.

**Response: **We appreciate all the comments and suggestions. We have addressed them all and hope that the revised version is an improved version of the manuscript.

 Respectfully;

 The corresponding author

# Reviewer report: Reviewer response for version 2

**DOI:** 10.5256/f1000research.179923.r376377

**Published:** 2025-04-14

**Authors:** Lex Bouter

**Affiliations:** 1 Vrije Universiteit Amsterdam, Amsterdam, North Holland, The Netherlands

The authors did a good job in responding to the comments made by the reviewers, including reframing of the research question. The revised manuscript is a substantial improvement of the report, but there are still a few issues which need attention.

- Some sentences are difficult to follow and there are still a few spelling and grammar errors in the manuscript. 
- Please completely remove language suggestive of causality (e.g. effect, affect, impact), as the study design doesn’t allow for a causal interpretation of the associations found.
- Please remove from the text the redundancy of providing both 95% CI and P values by removing all p-values from the manuscript when also a 95% CI is provided.
- One sentence in the abstract seems to be wrong: *However, gold OA model wasn’t significantly associated with Scopus Q1 ranking (OR, 0.49; 95% CI, 0.25-0.94; P=0.032), indicating that hybrid journals were more likely to have such ranking. *In the Results section you say about this: *…hybrid journals were associated with ranking in Q1 of Scopus as the odds of gold OA journals being ranked as Q1 was less than one (OR, 0.49; 95% CI 0.25–0.94; P = 0.032). *These statements contradict each other. I believe that the second one is correct, as the 95% CI doesn’t include 1. Furthermore, the sentence in the Results section refers to Table 4, but the table doesn’t contain the point estimate and 95% CI cited.
- The final sentence of the conclusion ( *Gold OA journals are encouraged to reduce their fees to facilitate global research access*) in the abstract is unrelated to the study results and should be removed.
- Table 2-4 are not very clear. Please explain whether the factors listed in the table are adjusted for the influence of the others. In other words: are these univariate or multivariate estimates? Furthermore, you specify only the reference category of the reported OR for the first factor in each table, but fail to explain what the reference category is for the other factors included.
- Not only the P values, but also the coefficients and standard errors are redundant in tables 1-4 and can be removed.
- In Table 3 the OR (95% CI) for the factor *journal published both clinical and basic sciences* is missing.
- The Discussion section contains a number of recommendations to lower subscription rates and APCs or to give waivers to specific groups. While I sympathize with these pleas, there is no ground for them in the outcomes of the study and they should be removed from the manuscript.

Is the work clearly and accurately presented and does it cite the current literature?

No

If applicable, is the statistical analysis and its interpretation appropriate?

Partly

Are all the source data underlying the results available to ensure full reproducibility?

No

Is the study design appropriate and is the work technically sound?

No

Are the conclusions drawn adequately supported by the results?

No

Are sufficient details of methods and analysis provided to allow replication by others?

No

Reviewer Expertise:

Research Integrity, Open Science, Methodology

I confirm that I have read this submission and believe that I have an appropriate level of expertise to confirm that it is of an acceptable scientific standard, however I have significant reservations, as outlined above.

# Response

**Published:** 2026-06-20

**Authors:** Abrar Thabit

**Affiliations:** Pharmacy Practice Department, King Abdulaziz University, Jeddah, Makkah Province, Saudi Arabia

Dear reviewer,

 We would like to thank you for taking the time to review and evaluate our manuscript. We have addressed all your comments and have revised the manuscript accordingly. An updated version of the manuscript highlighting the changes is attached. Please find below the responses to your comments.

 1. Some sentences are difficult to follow and there are still a few spelling and grammar errors in the manuscript.

**Response: **The manuscript underwent proofreading to correct the errors.

 2. Please completely remove language suggestive of causality (e.g. effect, affect, impact), as the study design doesn’t allow for a causal interpretation of the associations found.

**Response: **The language was toned down, and these words were removed where appropriate to avoid casual interpretation.

 3. Please remove from the text the redundancy of providing both 95% CI and P values by removing all p-values from the manuscript when also a 95% CI is provided.

**Response: **Done.

 4. One sentence in the abstract seems to be wrong: However, gold OA model wasn’t significantly associated with Scopus Q1 ranking (OR, 0.49; 95% CI, 0.25-0.94; P=0.032), indicating that hybrid journals were more likely to have such ranking. In the Results section you say about this: …hybrid journals were associated with ranking in Q1 of Scopus as the odds of gold OA journals being ranked as Q1 was less than one (OR, 0.49; 95% CI 0.25–0.94; P = 0.032). These statements contradict each other. I believe that the second one is correct, as the 95% CI doesn’t include 1. Furthermore, the sentence in the Results section refers to Table 4, but the table doesn’t contain the point estimate and 95% CI cited.

**Response: **We reviewed the two sentences. The first one was the correct one. So, we edited the one in the results section and rephrased it. The result is listed in Table 5 and Table 4.

 5. The final sentence of the conclusion (Gold OA journals are encouraged to reduce their fees to facilitate global research access) in the abstract is unrelated to the study results and should be removed.

**Response: **Removed as suggested.

 6. Table 2-4 are not very clear. Please explain whether the factors listed in the table are adjusted for the influence of the others. In other words: are these univariate or multivariate estimates? Furthermore, you specify only the reference category of the reported OR for the first factor in each table but fail to explain what the reference category is for the other factors included.

**Response: **The analysis was multivariable generalized linear model. This was clarified in the statistical analysis section of the methods. For the other factors it is either a yes or no (e.g., affiliation with an organization or lack of affiliation, either indexed in EMBASE or not, etc.). In case of linear regression, there’s no reference. We believe this is clear to the reader. Therefore, no changes were made.

 7. Not only the P values, but also the coefficients and standard errors are redundant in tables 1-4 and can be removed.

**Response: **After consulting a statistician and based on one of the comments by reviewer 3, we realized a misunderstanding in reporting the results of the analysis. Therefore, we removed the OR column since it was incorrect and kept the β coefficient column but replaced the standard error with 95% confidence intervals.

 8. In Table 3 the OR (95% CI) for the factor journal published both clinical and basic sciences is missing.

**Response: **We apologize for missing that. However, this whole column of OR is now removed.

 9. The Discussion section contains a number of recommendations to lower subscription rates and APCs or to give waivers to specific groups. While I sympathize with these pleas, there is no ground for them in the outcomes of the study, and they should be removed from the manuscript.

**Response: **We appreciate this comment. These parts were removed as suggested, but the calls for lower APCs in the literature was kept with appropriate citation of relevant references.

 Respectfully;

 The corresponding author

# Reviewer report: Reviewer response for version 1

**DOI:** 10.5256/f1000research.175295.r352795

**Published:** 2025-01-17

**Authors:** Marc-André Simard

**Affiliations:** 1 Universite de Montreal, Montreal, Québec, Canada

This article compares the journal impact of hybrid and gold OA biomedical journals based on two proprietary data sources, namely Web of Science and Scopus. This study shows that, while it varies on certain conditions, gold OA journals may not result in more citations.

 I think criticism of gold OA is warranted, but I think the approach used by the authors is misguided because it ignores the market forces that are at play as well as other sociological factors that hybrid journals benefit from (e.g. publisher and journal reputation, prestige, green OA, transformative agreements, etc.).

 I also believe that the introduction is too short or not detailed enough and fails to grasp some of the main issues related to the topic such as publication prestige, impact, preprints, more inclusive data sources (such as OpenAlex and Dimensions), the for-profit nature of the system, article processing charges, OA policies and community infrastructure, transformative agreements, etc. The article would benefit from a more in-depth literature review. OA is a fast-changing topic involving multiple actors which the introduction does not accurately reflect.

 I also find the title/research question (Are gold journals worth it?) to be confusing as the article never really investigate more than research impact, which is only one of the facets (and I would argue it is the shallowest) of what makes OA/open science a more ethical choice. Furthermore, this link between research impact and publishing in OA journals is based on highly controversial and flawed journal-based metrics such as the impact factor and citescore, which are not a good predictor of individual article impact because the distribution of citations among journal articles is highly skewed (see Larivière and Sugimoto, 2018 [Ref 3]). Another problem with the design is that it doesn’t not take into consideration the fact that hybrid OA articles can also be OA (via green or APC payment), and generally benefit from greater historical prestige than gold journals. Multiple studies have shown that green OA papers tend to receive more citations on average than other types of OA for that reason (e.g. Piwowar et al. 2018).

 In my opinion, the discussion/conclusion sections raise some valid points, but they are often too superficial and could use more depth and comprehension of the complex market forces at play. For instance, calling on gold OA publishers to lower APCs, while noble, is not a realistic or a sustainable approach because it doesn’t consider the for-profit nature of the system and how these actors are taking advantage of OA to generate more profits (e.g. see Butler et al. 2023 [Ref 2]). There is also very little criticism of the hybrid model (and no APC average, when previous research has shown that they are higher than gold), when it is arguably even worse than gold journals: their APCs are higher, they charge both readers and authors, and are also parts of unsustainable transformative agreements. Why not also call them out on reducing their APC/subscription/transformative agreement prices?

 Some more specific comments:

  

- The abstract makes the false assumption that hybrid and gold are the most popular open access models, when both green and diamond are essentially just as big as the other two. The main difference is that they are generally not-for-profit, so they don’t benefit from the same visibility or attention as the other two models.
- There are some minor false information or assumption about OA:
  - Green OA doesn’t necessarily mean embargos
  - Green OA and page charges: green OA is free for authors in the vast majority of cases.
  - Green OA is allowed by the vast majority of publishers
  - Not all journals ask for APCs. There are more than 25 000 diamond OA journals (e.g. see Khanna et al. 2023 [Ref 1] or the Directory of open access journals)

Ultimately, I appreciate what the authors were trying to accomplish with this study, but I do believe that some major flaws in the study design (e.g. focusing on journal-based metrics) and assumptions that were made (e.g. ignoring specific factors about hybrid OA) hold it back to the point where I cannot approve of this article.

Is the work clearly and accurately presented and does it cite the current literature?

No

If applicable, is the statistical analysis and its interpretation appropriate?

Yes

Are all the source data underlying the results available to ensure full reproducibility?

Yes

Is the study design appropriate and is the work technically sound?

No

Are the conclusions drawn adequately supported by the results?

Partly

Are sufficient details of methods and analysis provided to allow replication by others?

Partly

Reviewer Expertise:

open science, open access, bibliometrics, science of science

I confirm that I have read this submission and believe that I have an appropriate level of expertise to state that I do not consider it to be of an acceptable scientific standard, for reasons outlined above.

## References

[1] : Recalibrating the scope of scholarly publishing: A modest step in a vast decolonization process. *Quantitative Science Studies* .2022;3(4) : 10.1162/qss_a_00228912-930 10.1162/qss_a_00228

[2] : The oligopoly’s shift to open access: How the big five academic publishers profit from article processing charges. *Quantitative Science Studies* .2023;4(4) : 10.1162/qss_a_00272778-799 10.1162/qss_a_00272

[3] : Measuring Research.2018; 10.1093/wentk/9780190640118.001.0001 10.1093/wentk/9780190640118.001.0001

# Response

**Published:** 2025-03-26

**Authors:** Abrar Thabit

**Affiliations:** Pharmacy Practice Department, King Abdulaziz University, Jeddah, Makkah Province, Saudi Arabia

Dear Dr. Simard,

 We would like to thank you for taking the time to review and evaluate our manuscript. We have addressed all your comments and have revised the manuscript accordingly. An updated version of the manuscript highlighting the changes is attached. Please find below the responses to your comments.

 1. I think criticism of gold OA is warranted, but I think the approach used by the authors is misguided because it ignores the market forces that are at play as well as other sociological factors that hybrid journals benefit from (e.g. publisher and journal reputation, prestige, green OA, transformative agreements, etc.).

**Response: **Based on this comment and the comments made by reviewer 1, we have revised the manuscript to reflect the impact at the journal level, rather than the article level. Specifically, we commented in the discussion on the relation of the publishing model and their impact with journals’ reputation and prestige (lines 206-208 and 274-278).

 2. I also believe that the introduction is too short or not detailed enough and fails to grasp some of the main issues related to the topic such as publication prestige, impact, preprints, more inclusive data sources (such as OpenAlex and Dimensions), the for-profit nature of the system, article processing charges, OA policies and community infrastructure, transformative agreements, etc. The article would benefit from a more in-depth literature review. OA is a fast-changing topic involving multiple actors which the introduction does not accurately reflect.

**Response: **We have expanded the introduction to include many of the suggested topics in the reviewer’s comment (lines 59-62, 67, and 74-83).

 3. I also find the title/research question (Are gold journals worth it?) to be confusing as the article never really investigate more than research impact, which is only one of the facets (and I would argue it is the shallowest) of what makes OA/open science a more ethical choice. Furthermore, this link between research impact and publishing in OA journals is based on highly controversial and flawed journal-based metrics such as the impact factor and citescore, which are not a good predictor of individual article impact because the distribution of citations among journal articles is highly skewed (see Larivière and Sugimoto, 2018 [Ref 3]). Another problem with the design is that it doesn’t not take into consideration the fact that hybrid OA articles can also be OA (via green or APC payment), and generally benefit from greater historical prestige than gold journals. Multiple studies have shown that green OA papers tend to receive more citations on average than other types of OA for that reason (e.g. Piwowar et al. 2018).

**Response: **After the extensive revision that made to the manuscript, we have also revised the title to reflect the content and the consideration of the impact at the journal level rather than at the article level. We discussed the controversy regarding OA publishing based on the suggested reference (Sugimoto and Larivière) and another reference (Casadevall, et al) in the introduction (lines 78-83), as well as in the discussion (lines 272-278). Regarding the last point of considering that hybrid journals also have gold OA option, we have already acknowledged this as one of the limitations of our study (lines 284-287).

 4. In my opinion, the discussion/conclusion sections raise some valid points, but they are often too superficial and could use more depth and comprehension of the complex market forces at play. For instance, calling on gold OA publishers to lower APCs, while noble, is not a realistic or a sustainable approach because it doesn’t consider the for-profit nature of the system and how these actors are taking advantage of OA to generate more profits (e.g. see Butler et al. 2023 [Ref 2]). There is also very little criticism of the hybrid model (and no APC average, when previous research has shown that they are higher than gold), when it is arguably even worse than gold journals: their APCs are higher, they charge both readers and authors, and are also parts of unsustainable transformative agreements. Why not also call them out on reducing their APC/subscription/transformative agreement prices?

**Response: **We completely agree with the argument presented by the reviewer. Therefore, and to address this comment, we added to the introduction the concern regarding the high APCs of hybrid journals and the high prices of transformative agreements (lines 59-62). Additionally, we also added this argument and the call to reduce these fees by hybrid journals in the same paragraph that called gold OA journals to reduce their APCs in the discussion (lines 265-271).

 5. The abstract makes the false assumption that hybrid and gold are the most popular open access models, when both green and diamond are essentially just as big as the other two. The main difference is that they are generally not-for-profit, so they don’t benefit from the same visibility or attention as the other two models.

**Response: **We edited the sentence and removed “the most” before “common publishing models (line 2).

 6. There are some minor false information or assumption about OA:

- Green OA doesn’t necessarily mean embargos
- Green OA and page charges: green OA is free for authors in the vast majority of cases.
- Green OA is allowed by the vast majority of publishers
- Not all journals ask for APCs. There are more than 25 000 diamond OA journals (e.g. see Khanna et al. 2023 [Ref 1] or the Directory of open access journals).

**Response: **We corrected the information presented in the introduction regarding green OA and added appropriate citations (lines 33-38). We also added to the discussion a part discussing diamond OA and the number of journals adopting this model of publishing (lines 258-265).

 7. Ultimately, I appreciate what the authors were trying to accomplish with this study, but I do believe that some major flaws in the study design (e.g. focusing on journal-based metrics) and assumptions that were made (e.g. ignoring specific factors about hybrid OA) hold it back to the point where I cannot approve of this article.

**Response: **We appreciate the reviewer’s feedback. As mentioned above, we have extensively revised the manuscript to describe the results from the journal-level perspective. We also added to the discussion the arguments regarding the financial barriers posed by both gold OA and hybrid journals. We hope that these revisions have improved the manuscript and its credibility.

 Respectfully;

 The corresponding author

# Reviewer report: Reviewer response for version 1

**DOI:** 10.5256/f1000research.175295.r352802

**Published:** 2025-01-06

**Authors:** Lex Bouter

**Affiliations:** 1 Vrije Universiteit Amsterdam, Amsterdam, North Holland, The Netherlands

In this article 201 hybrid and 201 gold medical open access journals are compared in terms of several citation-based indicators. The findings indicate that gold open access journals have a slightly higher Impact Factor and are more likely to be in Quartile 1 of Scopus. Strangely that first key finding is not mentioned in the abstract, while the second finding is formulated in a confusing way (‘…wasn’t significantly associated..”).

 The narrative is somewhat difficult to follow, as there is a disconnect between the research question and the design of the study. The title (‘Is publishing gold open-access worth it?’) doesn’t state for whom this question will be answered. It turns out to boil down to the question whether the Article Processing Charge (APC) offers value for money in terms of impact from the author perspective. And this is where the disconnect comes in: the authors study impact at journal level, while authors will be interested in impact at article level. The problem is that indicators like the Impact Factor and other journal level indicators are poor predictors of the number of citations which a specific article in the journal at issue will get. There seems to be a growing consensus that gold open access publication leads to more citations and more citation diversity compared to paywalled publications. (See: Jeffrey Brainard. Open-access papers draw more citations from a broader readership. Science 24 January 2024. https://www.science.org/content/article/open-access-papers-draw-more-citations-broader-readership ).

 But there are more reasons why the study design is less suitable to answer the research question:

- Hybrid journals are a mix of gold open access and paywalled publications, which diminishes the contrast studied.
- There are many differences between the included hybrid and gold open access journals that render causal interpretations very difficult. A much better study design would consist of a comparison between gold open access publications to a carefully matched group of similar paywalled publications.
- No mention is made of open methods (preregistration, study protocol in public repository) or open data. Please repair or explain why this is not done.
- Some indicators studied seem to concern absolute counts of citations. This is strange as the total number of articles will differ substantially between journals.

Insufficient attention is given to the fact that from the perspective of authors both gold open access publications and paywalled publications suffer from financial barriers, as for both types of publications costs are involved that may be difficult to bear for authors or their institutions: APCs or journal subscriptions, respectively. The way forward might be diamond open access, but that promising solution is not mentioned in the article. The authors say that green open access involves page charges, which is not the case. (See e.g.: Open Access Network. https://open-access.network/en/information/open-access-primers/green-and-gold) Green open access (self-archiving), however, is correctly advocated as a way to make paywalled publications available for everyone, although that approach has a few disadvantages in terms of accessibility and sustainability.

 Finally, I sympathize with the plea to lower APC, but don’t see the connection to the findings of the study. In summary, the research question cannot be answered well with the study design at issue and already seems to be answered by others.

Is the work clearly and accurately presented and does it cite the current literature?

No

If applicable, is the statistical analysis and its interpretation appropriate?

Partly

Are all the source data underlying the results available to ensure full reproducibility?

No

Is the study design appropriate and is the work technically sound?

No

Are the conclusions drawn adequately supported by the results?

No

Are sufficient details of methods and analysis provided to allow replication by others?

No

Reviewer Expertise:

Research Integrity, Open Science, Methodology

I confirm that I have read this submission and believe that I have an appropriate level of expertise to state that I do not consider it to be of an acceptable scientific standard, for reasons outlined above.

# Response

**Published:** 2025-03-26

**Authors:** Abrar Thabit

**Affiliations:** Pharmacy Practice Department, King Abdulaziz University, Jeddah, Makkah Province, Saudi Arabia

Dear Prof. Bouter,

 We would like to thank you for taking the time to review and evaluate our manuscript. We have addressed all your comments and have revised the manuscript accordingly. An updated version of the manuscript highlighting the changes is attached. Please find below the responses to your comments.

**1. In this article 201 hybrid and 201 gold medical open access journals are compared in terms of several citation-based indicators. The findings indicate that gold open access journals have a slightly higher Impact Factor and are more likely to be in Quartile 1 of Scopus. Strangely that first key finding is not mentioned in the abstract, while the second finding is formulated in a confusing way (‘…wasn’t significantly associated..”).**

**Response: **We added the slight difference in IF; however, it was actually the hybrid journals that tended to have Q1 ranking in Scopus more than gold OA journals. We added both results to the abstract (lines 12-14).

**2. The narrative is somewhat difficult to follow, as there is a disconnect between the research question and the design of the study. The title (‘Is publishing gold open-access worth it?’) doesn’t state for whom this question will be answered. It turns out to boil down to the question whether the Article Processing Charge (APC) offers value for money in terms of impact from the author perspective. And this is where the disconnect comes in: the authors study impact at journal level, while authors will be interested in impact at article level. The problem is that indicators like the Impact Factor and other journal level indicators are poor predictors of the number of citations which a specific article in the journal at issue will get. There seems to be a growing consensus that gold open access publication leads to more citations and more citation diversity compared to paywalled publications. (See: Jeffrey Brainard. Open-access papers draw more citations from a broader readership. Science 24 January 2024. https://www.science.org/content/article/open-access-papers-draw-more-citations-broader-readership).**

**Response: **We highly appreciate the reviewer’s highlight and the differentiation between the article level vs. the journal’s level impact, particularly in terms of citation and the diversity of citations. While we did emphasize the impact of the model on the impact at the journal level, we have edited the claims that our findings have an impact at the article level and changed them to reflect the impact at the journal level. We have revised the manuscript, including the title, to reflect that our work focused on the impact at the journal level rather than at the article level (lines 74-83 in the introduction, as well as lines 152 and 220 in the discussion). We also cited the suggested article in the updated objective statement (line 78). In addition, we added to the discussion a paragraph on the differentiation between the two levels of impact and cited some of the studies mentioned in the article you kindly provided (lines 193-214).

**3. But there are more reasons why the study design is less suitable to answer the research question:**

- **Hybrid journals are a mix of gold open access and paywalled publications, which diminishes the contrast studied.**
- **There are many differences between the included hybrid and gold open access journals that render causal interpretations very difficult. A much better study design would consist of a comparison between gold open access publications to a carefully matched group of similar paywalled publications.**
- **No mention is made of open methods (preregistration, study protocol in public repository) or open data. Please repair or explain why this is not done.**
- **Some indicators studied seem to concern absolute counts of citations. This is strange as the total number of articles will differ substantially between journals.**

** Response: **Thank you for this extensive note. As in the case of any published research, limitations to the methodology can exist.

- We have already acknowledged the first point as one of the study limitations (lines 284-287).
- Regarding the second point, we did intend to include a random sample of journals. In order to control for the potential confounders and as an alternative to matching, we conducted multinomial logistic regression as described in the statistical analysis subsection of the methods. We added this justification to lines 128-129. However, we also added your point to the limitations as another way to control the confounders (lines 291-294).
- Regarding the third point, we added a clarification that only “peer-reviewed” journals (which are indexed in WOS and Scopus) were included. As such, the listed examples were excluded since they’re not typically indexed (lines 89-92).
- We agree that the total number of articles and the number of citations should be considered together, which was resolved by the advent of IF and CiteScore. We added a footnote to Table 1 regarding this issue. We also removed from the main text of the results “number of citations” to avoid highlighting it as one of the key findings. Lastly, we removed from the discussion the paragraph commenting on the difference in the total number of citations between the two groups (lines 229-233). We also added a relevant discussion on this matter to lines 272-278 of the discussion.

**4. Insufficient attention is given to the fact that from the perspective of authors both gold open access publications and paywalled publications suffer from financial barriers, as for both types of publications costs are involved that may be difficult to bear for authors or their institutions: APCs or journal subscriptions, respectively. The way forward might be diamond open access, but that promising solution is not mentioned in the article. The authors say that green open access involves page charges, which is not the case. (See e.g.: Open Access Network. https://open-access.network/en/information/open-access-primers/green-and-gold) Green open access (self-archiving), however, is correctly advocated as a way to make paywalled publications available for everyone, although that approach has a few disadvantages in terms of accessibility and sustainability.**

**Response: **We agree that we haven’t given diamond OA sufficient attention and emphasized its importance given the financial barriers associated with both gold OA and subscription-based publishing. Therefore and to fill this gap, we added to the introduction a sentence defining diamond OA (lines 32-33). We also edited the statements defining green OA in the introduction and added the appropriate citations including the website suggested by the reviewer (lines 33-38). Moreover, we added a part discussing diamond OA and its importance in overcoming the financial barriers to the discussion section in comparison to gold OA and subscription-based publishing (lines 258-265).

**5. Finally, I sympathize with the plea to lower APC, but don’t see the connection to the findings of the study. In summary, the research question cannot be answered well with the study design at issue and already seems to be answered by others.**

**Response: **After adding the part discussing diamond OA and that it is still growing with a very limited number of journals adopting it compared with the number of gold OA journals, we believe that the context and the flow of this paragraph in the discussion provides a justification for this plea (lines 247-271).

 Respectfully;

 The corresponding author
